# Effect of doctor–patient news-induced moral judgments on pain empathy for doctors and patients in China

**DOI:** 10.3389/fnins.2022.1037659

**Published:** 2022-11-23

**Authors:** HuiLing Li, Dong Zhao, BinJie Yang, YuHui Zhao, HanZhi Huang, Huan Jiang, MeiFen Chen, Qiang Zhou, Liang Ying

**Affiliations:** ^1^Department of Psychology, Wenzhou Medical University, Wenzhou, China; ^2^Renji College, Wenzhou Medical University, Wenzhou, China; ^3^School of Education, Wenzhou University, Wenzhou, China

**Keywords:** moral judgment, pain empathy, ERP, SVM, doctor–patient relationship

## Abstract

**Objective:**

Pain empathy’s preferential nature tends to trigger prejudice and intergroup conflicts. Given the current degree of proliferation of doctor–patient conflict news in China, this study aims to determine whether readers of doctor–patient news-initiated moral judgments prefer pain empathy for doctors or patients.

**Materials and methods:**

This study utilized localized doctor–patient news with high or low moral performance (based on morality ratings of patients’ behaviors) as moral-judgment-eliciting materials, and painful pictures as pain empathy-eliciting materials. The event-related potential (ERP) technique was utilized to assess moral judgment’s effect on the cognitive empathy component and to investigate electroencephalogram signals’ accuracy in classifying four brain response patterns when facing doctor or patient is experiencing or not experiencing pain.

**Results:**

Under low moral text material, participants exhibited smaller mean wave amplitude of positive 300 (P3) and late positive potential (LPP) to painful pictures than non-painful pictures when facing patients; under high moral text material, participants exhibited larger mean wave amplitude of P3 and LPP to painful pictures than non-painful pictures when facing doctors. Electroencephalogram (EEG) signals’ classification accuracy was significant in 0–1,000 ms in both high and low moral judgments, but the classification accuracy was higher in low moral judgments in some cognitive empathy stages (0.51, 0.53–0.55, 0.66–0.79, and 0.88–1 s).

**Conclusion:**

Under low moral judgment, individuals pay less attention to the patient’s (perpetrator’s) pain; under high moral judgment, individuals empathize with the doctor (the person praised), showing that news-induced moral judgment can sway readers’ empathy for different social groups. In cognitive empathy, individuals’ brain representations are more discriminatory under low than high moral judgments when confronted with pain by doctors and patients, which provides insight into objectively recognizing group bias.

## Introduction

Arguably, the doctor–patient relationship—that is, the interaction between the care provider and service recipient (Harbishettar et al., 2019)—is influenced by the general public’s empathy in response to the media coverage of doctor–patient conflicts (Stefanello, 2022). Currently, in China, the doctor–patient relationship is turbulent, and news covering doctor–patient conflicts is rapidly proliferating, predominantly focusing on negative reporting of patients harming doctors (Sun et al., 2018). Indubitably, some media outlets consciously broadcast news regarding harmonious doctor–patient relationships to compensate for the general one-sided reporting tendencies (Zhou et al., 2021); however, the measures implemented thus far have been either insufficient or ineffective (The Lancet, 2020).

Empathy is defined as an individual’s ability to experience others’ emotional states and mirror similar states in themselves (Michaels et al., 2014), which, in turn, enhances interpersonal communication efficiency (Singer and Lamm, 2009) and promotes individuals’ pro-social behavior (Wang et al., 2018; Smith et al., 2020). Pain empathy–a type of empathy–refers to the ability to empathize with observed as well as imagined pain (Fitzgibbon et al., 2010). Prior studies have demonstrated that pain empathy is preferential. For example, people exhibit stronger pain empathy for close friends than for strangers (Zhou et al., 2019), and for people with the same ethnicity than for those with another ethnicity (Fabi and Leuthold, 2018). Furthermore, people can distinguish the moral level of others’ behavior during pain empathy and exhibit higher pain empathy levels toward people who engage in ethical behavior (Cameron et al., 2022). A recent study demonstrated that people are more likely to subject strangers who are “harming others” to physical pain than strangers who are “not helping others.” This finding suggests that human pain empathy is influenced by moral judgments (Yang et al., 2022).

Moral judgment is about how “blameworthy” a behavior is, in other words, is to assess whether an act is blameworthy (Siegel et al., 2017). News-induced moral opinions precipitate empathy and facilitate individuals’ choice to empathize with different entities after making different moral judgments (Zaki, 2019b). However, empathy may pose a danger when the media use empathy to trigger bias and hatred (Bloom, 2017). Importantly, such biased empathy is narrow, and its unfairness makes people overlook those genuinely needing help, which, in turn, intensifies intergroup conflict (Fowler et al., 2021). Empathy that considers all people equal has greater social value and aids in the maintenance of social harmony and stability. Therefore, this study investigates the pain empathy mechanism after individuals’ exposure to common doctor–patient conflict news, to reveal how such news’ selection and description may reduce public prejudice against doctor or patient groups, which can help improve doctor–patient relationships.

Unfortunately, existing commonly used subjective assessments are time-intensive and fail to detect progressive changes in pain empathy. Moreover, human subjective ratings’ sensitivity is inadequate to identify the empathy process’ specific nuances and underlying mechanisms (Xiao et al., 2016). By contrast, utilizing event-related potential (ERP) techniques with high temporal resolution refines pain empathy’s neural mechanisms in more naturalistic contexts (Coll, 2018). Pain empathy is categorized into bottom-up emotional and top-down cognitive empathy (Kopiś et al., 2020). The former represents the ability to spontaneously experience others’ inner emotions. Prior research has indicated that N1, N2 can distinguish between pain and non-pain stimuli and can be considered a marker of the automatic activation of emotionally salient stimuli (Li et al., 2020), however, a recent meta-analysis has shown that N1, N2 may not be a reliable index of pain empathy (Coll, 2018). The latter refers to the analysis and understanding of others’ internal states and involves stimuli processing rooted in knowledge and experience (Fan and Han, 2008; Ibáñez et al., 2011). This induces late ERP components, such as positive 300 (P3) and late positive potential (LPP), thereby triggering the cognitive evaluation of others’ pain (Coll, 2018). Therefore, we used P3 and LPP as late-ERP components. Further, explicit moral appraisals were found to induce LPP which is related to emotion, cognition, and motivation (Yoder and Decety, 2014). Thus, the following questions arise: Given that moral judgments are rooted in later cultivation (Kim and Park, 2019), do moral judgments influence cognitive empathy which is also based on later cultivation (the P3 and LPP components)?

Although empathy measurement is progressing, decoding human empathic perceptions, expressions, and behavior through the measurement results is relevant, but still in its infancy. Moreover, simultaneous advances in signal processing and machine learning techniques enable researchers to identify empathy in multimodal data and, consequently, provide objective assessments (Xiao et al., 2016). Machine learning has been applied to empathy classification and prediction, and patterns of resting-state fMRI connectivity of resonance and control networks have been shown to predict trait empathic concern (EC) (Christov-Moore et al., 2020), empathic ingroup preferences translating into behavior, and neural activation in their associated regions predicting pro-social behavior (Hein et al., 2010; Christov-Moore et al., 2017). The first single machine learning model for neural activation on pain empathy for comparing ingroups and outgroups was developed by Vaughn et al. (2018). This model showed that human empathic classification of ingroups and outgroups was 72% accurate, thus postulating that neural networks are sufficient and necessary conditions to distinguish between ingroups and outgroups (Vaughn et al., 2018). People tend to classify those with desirable moral performance as ingroup (Van et al., 2012); hence, individual neural responses’ ability to distinguish between doctor or patient pain under doctor–patient news-induced moral judgments can lend a theoretical basis for neural responses to predict pain empathy group preferences and provide criteria for group bias diagnosis.

Therefore, we collated localized news materials to elicit moral judgments, used pain-picture materials to elicit pain empathy, and collected ERP data. Through ERP analysis, we explored high vs. low moral judgments’ electrophysiological effects on cognitive empathy between doctor and patient groups during pain empathy. Through support vector machines (SVMs), we explored whether electrophysiological responses are differentiated in the treatment of patients vs. doctors experiencing or not experiencing pain, and if so, whether the degree of this differentiation is influenced by moral judgment.

## Materials and methods

### Research design

**FIGURE 1:**
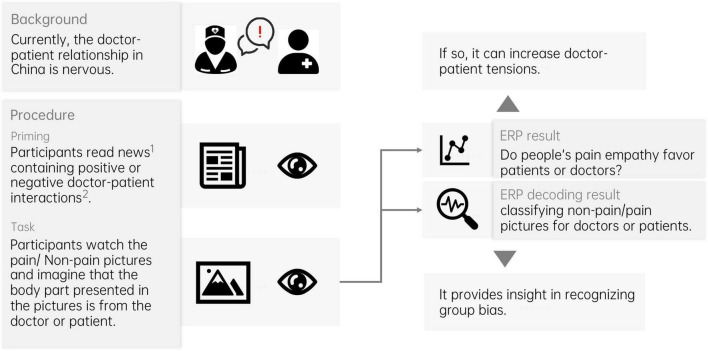
The research structure. ^1^News materials are selected through a rigorous scoring process. ^2^The degree of positive or negative doctor–patient interaction is determined by the level of morality exhibited by the patient’s behavior.

This study used a three-factor mixed design of 2 (high moral text material/low moral text material) × 2 (imagined subject as doctor/imagined subject as patient) × 2 (painful pictures/non-painful pictures); herein, high and low moral ratings were between-subject factors, and the remainder were within-participant factors. This study was approved by the Ethics Committee of Wenzhou Medical University (2022-017). All participants voluntarily participated in the study and signed an informed consent form. Participants were informed that they could withdraw from the study at any time if they no longer intended to participate. The research structure is shown in Figure 1.

### Participants

The sample size was calculated using G-Power, which indicated that 24 participants (Effect size *f* = 0.25, α = 0.05, 1-β = 0.8, number of groups = 2, number of measurements = 4, Corr. among rep measures = 0.5, non-sphericity correction = 1) were required; eventually, 30 participants were included (20 women). The mean age was 21.23 (SD = 2.42) years. All the patients were right-handed and exhibited normal or corrected visual acuity.

### Materials

Commonly used search engines in China (Baidu and Sogou) were used to retrieve 30 news articles. The keywords used were “doctor–patient,” “conflict,” “news,” and “relationship.” Two articles having excessively numerous words were excluded, the remained 28 textual materials had an average length of 509.39 ± 85.72 words each. Overall, the 28 textual materials were randomly divided into four groups, and 122 participants (61 women) who did not participate in the formal experiment were recruited and randomly divided into four groups as well. The mean age was 21.48 (SD = 0.94) years. Each group of textual materials was rated on a scale of 1–9 by one of the groups of participants. The rating dimensions were as follows: patient moral level (1 = low moral level, 9 = high moral level), pleasantness (1 = very unpleasant, 9 = high pleasantness), arousal (1 = fairly calm, 9 = fairly agitated), and dominance (1 = being dominated, 9 = dominating). The three highest and lowest moral-level patient ratings were selected as high moral text initiation material (*M* = 8.32, SD = 1.03) and low moral text initiation material (*M* = 1.21, SD = 0.49), respectively, *t* (119) = 114.23, *p* < 0.001 (see Appendix A for further details).

We used 40 fully scored pain and non-painful pictures (Meng et al., 2012) as pain-empathy-inducing stimuli. The painful pictures’ content included injury to a human body part–such as cutting or needling; the non-painful pictures’ content replaced the previous injury-related content–for example, replacing the needle with a cotton swab. All pictures were close to those of daily life, easy to understand, and unambiguous (see Appendix B for details on the material).

The Interpersonal Reactivity Index-C (IRI-C) was used to measure participants’ empathic abilities. The original version was developed by Davis in 1980, and the Chinese version was developed by Chan in 1987. The scale comprises four dimensions: perspective-taking (PT), fantasy (FS), EC, and personal distress (PD). The IRI-C exhibits a split-half reliability of 0.734, test–retest reliability of 0.737, and a variance rate of 46.342%, across the questionnaire’s four factors (Meng et al., 2012). Its reliability is good, and it demonstrates considerable validity in measuring participants’ empathy.

### Procedure

The experimental environment was a standard electroencephalogram (EEG) laboratory with soundproofing and no external noise disturbance. The light was bright and soft, and the room temperature was appropriate. The participants’ eyes were approximately 80 cm away from the computer screen, and their viewing angle was less than 10°.

Prior to conducting the formal experiment, the participants were prompted to adjust their sitting posture and place their hands on the corresponding keys on the keyboard. To prevent artifacts in the electrophysiological data, they were asked to avoid making any sound and refrain from moving body parts irrelevant to the experimental response.

The experimental procedure comprised an exercise and formal experiment. The exercise involved the random presentation of 20 pictures from the Pain Empathy Gallery (Meng et al., 2012); There are two types of pictures, pain and non-pain. The participants were asked to identify the types of pictures by pressing the “F” or “J” picture. Each identification provided correct, incorrect, or no response feedback to help participants become familiar with the keystrokes of the formal experiment. None of the pictures presented during the exercise were presented in the formal experiment.

The experimental procedure was divided into two categories–high and low ethics–according to the content of the priming text materials. Each block began with a short text on the doctor–patient relationship in which participants were asked to read at their own speed.

After reading, participants were instructed to rate patient moral level (1 = low moral level, 9 = high moral level) and then perform an imagery task when pictures were presented, imagining that the body part in the stimulus presented next belonged to the doctor or patient in the text. The imagined subjects were randomly prompted before each of the 40 trials with either “Please imagine that the part presented in the picture is a doctor in the text” or “Please imagine that the part presented in the picture is a patient in the text.”

**FIGURE 2:**
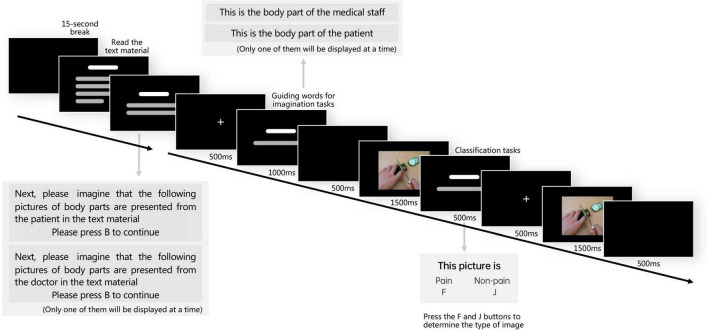
Flow diagram of experimental procedure. The experiment had two parts: reading text and viewing pictures; the low moral judgment group read low moral text and the high moral judgment group read high moral text.

To ensure active attention to the content of the pictures during the experiment, the participants were asked to classify the presented stimuli as pain or non-painful pictures by pressing the key “F” or “J” in keyboard. The correct rate of classification was used to determine whether the participants could understand the content of the pictures, and the stimulus pictures were presented twice to ensure that participants are able to notice the picture content. The experimental procedure is shown in Figure 2.

### Statistical analysis

#### Event-related potential data recording

The EEG data were recorded using a 64-channel EEG analysis system (ANT Neuroscan). The average value of the bilateral mastoid positions was used as a reference, with a filtered bandpass of 0.1–30 Hz and a sampling frequency of 500 Hz. The scalp impedance of each electrode was less than 5 kΩ.

#### Event-related potential data analysis

The ERP data were processed offline using EEGLAB 8.30 and ERPLAB 8.30 in MATLAB 2020b. The average value of the bilateral mastoids was taken as a reference, and horizontal electrooculography was automatically corrected. Waveform data with amplitudes greater than ±80 μV were considered artifacts and were rejected. The 200 ms before stimulus presentation was used as the baseline, 1,000 ms after stimulus presentation was used as the response period, and the analysis duration was 1,200 ms.

Based on the aim of this study and previous studies (Campanella et al., 2002; Hajcak et al., 2007; Meng et al., 2012; Cheng et al., 2017), P3 (350–450 ms) and LPP (time window of 400–700 ms) was selected as the late component of empathic pain processing. The electrode sites selected for the P3 and LPP components were FCz, Cz, Pz, C3, C4, Cp3, and Cp4 (distributed in the parietal lobe and near the central region). A four-factor repeated measures ANOVA with 2 (textual material moral level: high moral/low moral) × 2 (imagined subject: medical care/patient) × 2 (picture type: painful pictures/non-painful pictures) × 7 (electrode points: FCz, Cz, C3, C4, Pz, Cp3, and Cp4) was performed for the mean wave amplitude and peak latency, respectively, using SPSS 23.0.

#### Event-related potential decoding

Commonly used machine learning methods to evaluate empathy include linear regression, SVMs, and dynamic Bayesian networks. SVMs (Xiao et al., 2016) are a class of generalized linear classifiers that perform binary classification of data in a supervised learning manner. Its decision boundary is the maximum-margin hyperplane of the learned sample solution, which is powerful in its ability to learn data classification patterns with balanced accuracy and reproducibility. SVM has become a widely used classification tool with high generality and has been extended to several data science scenarios (Pisner and Schnyer, 2020).

Using SVM, we performed time-point-by-time quadratic decoding of high- and low-moral text material groups characterized by mean wave amplitude, that is, imagining pain or non-painful pictures where the subject was a doctor or a patient and derived decoding performance above the time point of chance (random level of 0.25).

First, a three-dimensional data matrix was obtained for each participant, including trial (240 trials per category) and electrode location (62 scalp positions) dimensions. After traversing all participants’ data, the low moral judgment group of 14 people was assembled into four dimensions (14, 240, 62, and 850), and the high moral judgment group of 16 people was assembled into four dimensions (16, 240, 62, and 850), corresponding to the label of each trial.

Decoding is then performed point-by-point using the function named “tbyt_decoding_kfold” under the decoding module of NeuroRA (Lu and Ku, 2020). The five trials of each class were averaged and decoded every five time points, followed by 10 triple-fold cross-validations. In the threefold cross-validation, 2/3 of the randomly selected trials were used to train the classifier, and the remaining 1/3 of the trials were used to evaluate the performance of the classifier. The evaluation process in each case was that the trained classifier predicts which of the four categories is the label of the test set at the current time point. Finally, the results were smoothed.

The function named “plot_tbyt_decoding” under the decoding module of NeuroRA was used for plotting. The cluster-based permutation test was used to determine whether there were differences in the mean wave amplitudes of different picture types for different imagined subjects. If differences existed then the time series of these differences were analyzed with the time interval set to 0.01 s, the time range from −0.2 to 1.0 s, and *p* set to.05.

After decoding the two groups separately on a time-point-by-time basis, an independent samples *t*-test was used to analyze whether there was a significant difference in the classification accuracy between the two groups of high and low moral text materials at each time point.

## Results

### Moral ratings

The results of the ratings of patients’ moral level showed that the independent sample *t*-test for high moral news (*M* = 8.25, SD = 1.06) vs. low moral news (*M* = 1.24, SD = 0.53) was *t*(88) = −38.73, *p* < 0.001, indicating that the participants were able to significantly distinguish between the moral levels of the two types of news.

### Interpersonal Reactivity Index-C

Table 1 reveals no significant difference between the high and low moral text material groups in terms of empathy in general and no significant difference in terms of different dimensions of empathy.

**TABLE 1:** The Interpersonal Reactivity Index-C result.

Dimension	High morality (M ± SD)	Low morality (M ± SD)	*t*	Sig.
Perspective taking	19.29 ± 4.30	18.07 ± 3.47	0.82	0.42
Fantasy scale	17.64 ± 2.27	18.71 ± 2.33	−1.23	0.65
Empathy concern	18.14 ± 2.25	19.36 ± 1.82	−1.57	0.21
Personal distress	15.14 ± 3.68	16.64 ± 3.18	−1.16	0.71
Total	54.64 ± 10.56	57.21 ± 11.74	−0.61	0.92

### Accuracy and reaction time

The accuracy rates and reaction times for each condition are presented in Table 2. All participants had significantly higher correct response rates for the picture types, indicating that they could effectively discriminate between the two picture types. The ANOVA results for reaction time showed a significant interaction between textual morality level and picture type (*F* (1, 29) = 1.049, *p* < 0.001, η^2^ = 0.306). Simple effects analysis indicated that the reaction time for looking at painful pictures after reading high morality news (*M* = 418.596, SD = 5.171) was shorter than that for non-painful pictures (*M* = 465.459, SD = 5.373). The reaction time to look at painful pictures after reading low moral text material was shorter (*M* = 410.588, SD = 5.680) than that for non-painful pictures (*M* = 434.068, SD = 6.663). This is consistent with previous research (Cui et al., 2016; Cheng et al., 2017; Galang et al., 2017), people will tend to prioritize painful pictures that contain threat-related information (Clauwaert et al., 2019).

**TABLE 2:** Accuracy and reaction time result.

Types of text material	Imagine subject	Picture type	ACC (M ± SD)	RT (M ± SD)
High morality	Doctor	Painful	0.96 ± 0.03	428.54 ± 154.72
		Non-painful	0.91 ± 0.06	476.89 ± 166.12
	Patient	Painful	0.96 ± 0.05	408.65 ± 145.22
		Non-painful	0.94 ± 0.07	454.03 ± 166.5
Low morality	Doctor	Painful	0.95 ± 0.04	405.39 ± 177.34
		Non-painful	0.91 ± 0.07	446.15 ± 178.79
	Patient	Painful	0.90 ± 0.09	415.79 ± 170.33
		Non-painful	0.94 ± 0.05	421.99 ± 185.01

### Electrophysiological data

#### Positive 300

The mean wave amplitude main effect of textual material morality level (*F* (1, 29) = 37.209, *p* < 0.001, η^2^ = 0.045), imagined subject (*F* (1, 29) = 5.817, *p* = 0.016, η^2^ = 0.007), picture type (*F* (1, 29) = 9.312, *p* = 0.002, η^2^ = 0.012) was significant. Lower morality level (*M* = 2.845, SD = 0.173), imagining doctor (*M* = 2.407, SD = 0.168), non-painful picture (*M* = 2.483, SD = 0.168) induced a greater wave amplitude than higher morality level (*M* = 1.397, SD = 0.162), imagining patient (*M* = 1.835, SD = 0.168), and painful picture (*M* = 1.759, SD = 0.168).

The mean wave amplitude interaction of the moral level of textual material, imagined subject, and picture type was significant, *F* (1, 29) = 4.914, *p* = 0.027, η^2^ = 0.006; Pairwise comparison showed that after reading low morality materials, participants had smaller amplitudes of patients’ pain pictures (*M* = 1.882, SD = 0.347) than non-pain pictures (*M* = 3.242, SD = 0.324); after reading high morality materials, had greater amplitudes of doctors’ pain pictures (*M* = 2.258, SD = 0.347) than non-pain pictures (*M* = 1.116, SD = 0.324). No other analysis yielded significance.

The peak latency main effect of picture type (*F* (1, 29) = 3.957, *p* = 0.047, η^2^ = 0.005) was significant, painful picture (*M* = 542.902, SD = 3.518) induced longer latency than non-painful picture (*M* = 534.823, SD = 3.527). No other effect reached significance.

#### Late positive potential

The mean wave amplitude main effect of textual material morality level (*F* (1, 29) = 11.824, *p* = 0.001, η^2^ = 0.015), imagined subject (*F* (1, 29) = 4.527, *p* = 0.034, η^2^ = 0.006), picture type (*F* (1, 29) = 8.553, *p* = 0.004, η^2^ = 0.011) was significant. Lower morality level (*M* = 2.546, SD = 0.174), imagining doctor (*M* = 2.390, SD = 0.168), and non-painful picture (*M* = 2.485, SD = 0.168) induced a greater wave amplitude than higher morality level (*M* = 1.729, SD = 0.162), imagining patient (*M* = 1.885, SD = 0.168), and painful picture (*M* = 1.790, SD = 0.168).

The mean wave amplitudes at the different electrode sites differed significantly (*F* (1, 29) = 3.619, *p* < 0.05, η^2^ = 0.024): CP4 (*M* = 1.460, SD = 0.315) than FCz (*M* = 2.878, SD = 0.315), Cz (*M* = 2.996, SD = 0.315), C3 (*M* = 2.345, SD = 0.315), and C4 (*M* = 2.477, SD = 0.315) were lower; Pz (*M* = 1.112, SD = 0.315) was lower than FCz (*M* = 2.878, SD = 0.315), and Cz (*M* = 2.996, SD = 0.315), C3 (*M* = 2.345, SD = 0.315), and C4 (*M* = 2.477, SD = 0.315) were low. The interaction between text material morality level, imagined subject, and picture type was significant, *F* (1, 29) = 4.889, *p* = 0.027, η^2^ = 0.006. Pairwise comparison showed that after reading low morality materials, participants had smaller amplitudes of patients’ pain pictures (*M* = 1.620, SD = 0.347) than non-pain pictures (*M* = 2.959, SD = 0.347); after reading high morality materials, had greater amplitudes of doctors’ pain pictures (*M* = 2.529, SD = 0.325) than non-pain pictures (*M* = 1.426, SD = 0.325). No other effect showed any significance.

Peak latencies differed significantly at the morality level (*F* (1, 29) = 13.460, *p* < 0.001, η^2^ = 0.017), longer for those initiated by high moral text material (*M* = 548.0, SD = 3.407) than for low moral text material (*M* = 529.725, SD = 3.634). No other effect reached significance.

**FIGURE 3:**
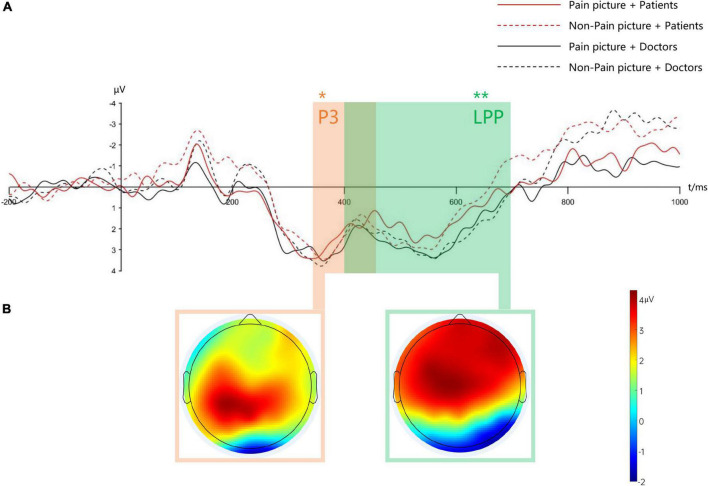
Mean waveform and mean topographic map under low moral text material. Panel **(A)** shows the mean waveforms of positive 300 (P3) and late positive potential (LPP) (selected electrode points are FCz, Cz, Pz, C3, C4, CP3, CP4), respectively, * indicates *p* < 0.05; ** indicates *p* < 0.01; panel **(B)** shows the mean topographic map of P3 (350–450 ms) and LPP (400–700 ms) for a specific time window.

The total mean and topographic maps of ERP evoked by pain and non-pain stimuli under the initiation of high moral text material and low channel text material are shown in Figure 3.

### Correlation analysis

A correlation analysis was done between IRI-C scores and the mean wave amplitude of P3 and LPP to painful pictures, and it was found that IRI-C scores were positively correlated with the mean wave amplitude of P3 (*r* = 0.148, *p* < 0.01) and LPP (*r* = 0.121, *p* < 0.05) to painful pictures, i.e., the stronger the empathic ability of the participants, the higher the wave amplitude of P3 and LPP to painful pictures.

### Event-related potential decoding data

**FIGURE 4:**
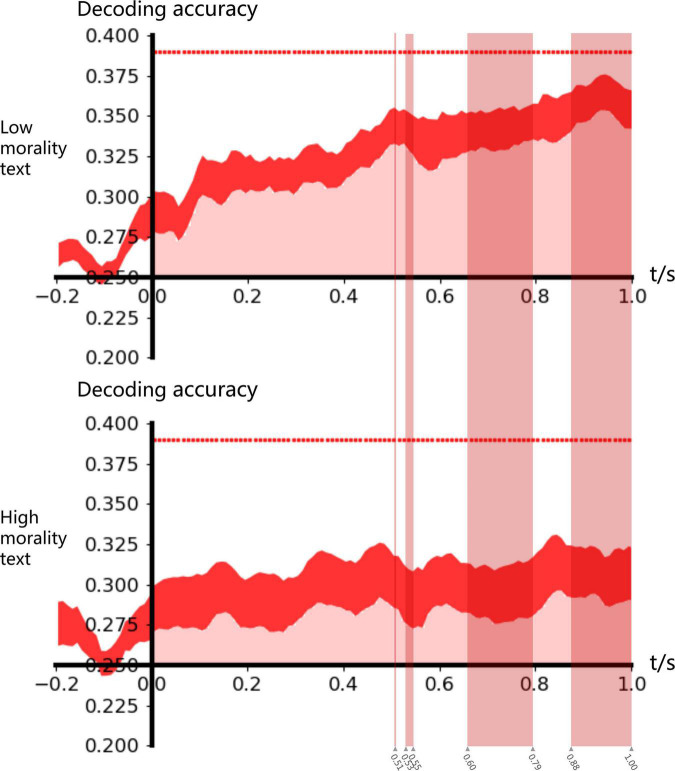
ERP decoding accuracy. In 0–1 s, the classification accuracy was significantly greater than the random classification level of 0.25. In 0.51 s, 0.53–0.55 s, 0.66–0.79 s, and 0.88–1 s, the classification accuracy under the influence of high moral text was significantly greater than that under the influence of low moral material (*p* < 0.05).

The cluster-based permutation test suggested that both high and low ethical text material groups were significant in 0–1.0 S; an independent samples *t*-test found that the low ethical evaluation group’s classification accuracy was significantly greater than that of the high ethical evaluation group in 0.51 S, 0.53–0.55 S, 0.66–0.79 S, and 0.88–1.00 S, as shown in Figure 4.

## Discussion

This study investigated the effect of doctor–patient relationship news on pain empathy in readers. Textual material at different moral levels was used to initiate participants’ moral judgments, and an imagery task was interspersed to allow participants to empathize with pain for specific objects in the textual content.

Unexpectedly, this study found that after making low moral judgments of patients, individuals had lower P3 and LPP wave amplitudes for the patient’s pain than in the absence of pain. And individuals had longer peak latencies in LPP after reading high moral material than after reading low moral material. Studies have shown that after some things induce negative emotions, people’s pain empathy level decreases, and the neural representation of empathy when observing others’ pain is inhibited to a certain extent, such as participants have significantly smaller parietal P3 amplitudes for painful pictures than neutral pictures. This is because individuals divert their own attention from previous pain experiences by reducing their attention to other people’s pain stimuli and increasing their attention to neutral stimuli that are not related to pain (Li et al., 2017; Fan et al., 2021). As negative news, low-morality text materials may induce people’s anger, sadness and other negative emotions to a certain extent. Individuals have already experienced pain during the reading process. After that, they are unwilling to bear more pain, as a result, they pay less attention to pain stimuli.

Moreover, both P3 and LPP are neural indicators for the stage of cognitive processing and evaluation of others’ pain (Fan and Han, 2008; Yoder and Decety, 2014). Behaviors reflecting low morality have been shown to reduce the LPP amplitude (Chiu Loke et al., 2011), suggesting that moral judgment may have an impact on the top-down processing of pain empathy and that patients’ antisocial behavior reduces pain empathy in readers. Therefore, readers may avoid pain stimuli after reading sad news with low morality, and the patient’s harmful behavior may further reduce the readers’ attention to the patient’s pain. Worse yet, readers may even think that the patient should suffer from pain, thus causing even lower P3 and LPP amplitudes for patients suffering from pain than for neutral stimuli.

Another gratifying finding was that after reading high-morality news, individuals had higher amplitudes of P3 and LPP for doctors’ painful picture than those non-painful, indicating that individuals developed pain empathy for doctors. In high-morality news, both doctors and patients are subjects with positive images: Patients are grateful; doctors are positively recognized and highly commended by patients. Individuals may choose to empathy for higher-morality subjects through moral judgment (Smith and Frieze, 2003; Kirmani et al., 2017). Recently, a growing number of research works have focused on how objects’ moral level affects participants’ empathy for them. Compared with self-inflicted AIDS patients, people will show stronger empathy and willingness to donate to innocent AIDS patients (Guo et al., 2022); a fair person in pain causes increased activation of a brain region involved in empathy in the observer compared to an unfair person, for example, individuals have stronger P3 empathy responses to individuals who appear to be trustworthy, and even have negligible empathy responses to individuals who appear to be untrustworthy (Sessa and Meconi, 2015). Thus, moral judgments can facilitate the selection of appropriate social subjects to avoid social losses such as betrayal and deception by engaging with higher moral objects. In the process of empathy, the preference for “good people” stems from self-preservation, because people learn later in their upbringing that “good people” are less likely to betray them.

In summary, moral judgment influence pain empathy; however, do individuals respond differently to neuropathic pain empathy from different groups? In other words, is it possible that different brain response patterns characterize pain empathy differently between groups?

Individuals did not empathize with others indiscriminately, and the accuracy of individuals’ classification of doctors or patients suffering from pain or not, was significantly greater than random levels on the whole-brain wave amplitude under high and low moral judgments. This indicates that individuals behave differently when they empathize with different groups suffering from pain. Individual empathy is moderated by group identity, such as social rank, and some researchers have established different social ranks by having participants perform a dot estimation task and by informing them of their achievement levels. The results showed that people had lower levels of pain empathy for higher-ranked individuals (Feng et al., 2016). In fact, people’s empathy process is based on a social categorization model, i.e., they identify the social group to which the empathic target belongs during the empathy process (Read, 2021). In the present study, part of the reason for participants’ differential empathy for doctors and patients may be the natural social identity division between doctors and patients.

Notably, the classification accuracy was higher under low moral judgments than high moral judgments in the later components of ERP, suggesting that individuals’ cognitive processing and evaluation of others’ pain resulted in “differential” pain empathy for groups with different moral performance, supported by neural network patterns. Differences between explicit and implicit moral judgments exist mainly in the later stages of moral processing such as P3 and LPP (Zhan et al., 2018). This distinction is due to both the difference in social identity and the difference in assessment results at the moral level. Neural networks may be sufficient and necessary to distinguish between different groups (Vaughn et al., 2018), which may be equally applicable in pain empathy, suggesting that machine learning classification of brain representations could be used both as an intervention to reduce group bias and an objective diagnostic tool.

In general, news is a double-edged sword, individual pain empathy for doctors and patients is influenced by news-induced moral assessments to these two groups. People are more likely to empathize with ingroups than outgroups, and people may classify high moral individuals as ingroups and low moral individuals as outgroups based on their moral assessment of a person (Van et al., 2012), which may be the reason why moral assessments can trigger “differential” or even “biased” pain empathy. In this study, two representative news stories, “Patients Reward Doctors” and “Patients Harm Doctors,” were selected. In the former, the doctor is a helper and commended by patients, which leave a good impression on the audience. In the latter, the patient is a perpetrator, which leads to indifference or even schadenfreude to the patient’s pain. The doctor will feel respected and thus behave better; the patient may break down, which may exacerbate the disconnect between doctors and patients to some extent (Zaki, 2019a). When reporting, the media should weigh the positive and negative behaviors of doctors and patients. They should not label one as a victim or a victimizer to create public attention, gain more views, or generate traffic, and should instead clearly declare the objective conflict of interest between doctors and patients (Al-Balushi, 2020).

This study only selected two representative types of doctor–patient news articles and moral judgment available was only for patients. Currently, most negative doctor-patient news articles depict patients causing physical harm to doctors, so most of the low-morality textual material in this experiment shows doctors who have been physically harmed, and such content may promote public empathy for doctors’ pain. In the future, more ecological validity studies can be conducted from the perspectives of adding more types of doctor–patient news, expanding moral judgment to different subjects, producing globalized news materials, and further building mathematical models with large data samples to predict people’s empathy for different groups after reading different moral performance news.

## Conclusion

After low moral judgment of a patient, the individual is less concerned when the patient suffers pain; in the text where the doctor is thanked by the patient, the individual develops pain empathy for the doctor. Individuals’ pain empathy for doctors and patients was more differentiated in “acquired” cognitive processing after low moral judgments of patients than after high moral judgments.

Individuals’ pain empathy for the doctor–patient group was differentiated and moral judgments may have guided their biases.

## Data availability statement

The datasets presented in this study can be found in online repositories. The names of the repository/repositories and accession number(s) can be found in the article/Supplementary material.

## Ethics statement

The studies involving human participants were reviewed and approved by the Ethics Committee of Wenzhou Medical University (2022-017). The patients/participants provided their written informed consent to participate in this study.

## Author contributions

HL, DZ, BY, and LY developed the original idea and the protocol and drafted the manuscript. YZ, HH, and HJ abstracted and analyzed data and improved the manuscript. HL, MC, QZ, and LY contributed to the critical revision of the manuscript for important intellectual content. MC provided guidance on research topics and design. All authors contributed to the article and approved the submitted version.
