# Supplementary material for: Effect of doctor–patient news-induced moral judgments on pain empathy for doctors and patients in China
Source: Front Neurosci. 2022 Nov 23;16:1037659. doi: 10.3389/fnins.2022.1037659 (PMC9726916; doi:10.3389/fnins.2022.1037659)
Supplement: Supplementary file 1 [file Data_Sheet_1.docx]

| Num. | | text | | word count | patient moral level | | | | pleasantness | | | | arousal | | | | | dominance | | | |
| --- | --- | --- | --- | --- | --- | --- | --- | --- | --- | --- | --- | --- | --- | --- | --- | --- | --- | --- | --- | --- | --- |
|  |  |  |  |  | M | SD | | | M | | SD | | M | | SD | | | M | | SD | |
| 1 | | 6月2日4点多，湖南一医院发生暴力伤医事件。一名肺癌并多发转移患者因抢救无效死亡，参与抢救的值班女医生和一名怀孕5个半月的值班护士被家属暴力殴打致伤，家属还逼值班医生下跪，对其实施殴打。目前公安部门已成立暴力伤医事件专案组，正在调查取证。 据湖南省卫生厅介绍，王医生被患者家属殴打致颅脑损伤：脑震荡、头皮血肿、创伤性蛛网膜下腔出血，多发性软组织挫伤，耳鸣、听力下降，外伤性耳聋，外伤性子宫损伤。谭护士因被患者家属殴打恐吓导致晚期先兆流产。目前两名医务人员正在医院住院接受治疗。 在此过程中，死者家属还多次公然侮辱、恐吓医务人员，打砸医生办公室，殴打医院保安，在病房烧、撒纸钱，点蜡烛，直到2日下午5点才将死者尸体移离病房，严重扰乱了医院正常的医疗秩序。  At 4 o 'clock on June 2, a violent incident occurred in a hospital in Hunan Province. A patient with lung cancer and multiple metastases died due to ineffective rescue. The female doctor on duty who participated in the rescue and a nurse on duty who was five and a half months pregnant were violently beaten by their family members, who also forced the doctor on duty to kneel and beat them. At present, the public security department has set up a task force to investigate and collect evidence.  According to the Hunan Provincial Health Department, Wang was beaten by the patient's family members and caused brain injuries: concussion, scalp hematoma, traumatic subarachnoid hemorrhage, multiple soft tissue contusion, tinnitus, hearing loss, traumatic deafness, and traumatic uterine injury. Nurse Tan was beaten and threatened by the patient's family which resulted in her late threatened abortion. Two medical personnel are currently hospitalized and receiving treatment.  In this process, the family members of the deceased also repeatedly insulted and threatened medical staff, smashed the doctor's office, beat the hospital security, burned and scattered joss money in the ward, lit candles, and did not move the body of the deceased out of the ward until 5 PM on the 2nd, seriously disrupting the normal medical order of the hospital. | | 215 | 1.19 | .477 | | | 1.42 | | .672 | | 6.61 | | 2.076 | | | 4.68 | | 1.777 | |
| 2 | | 上海一医院心胸外科发生了一起极为恶劣的严重伤害无辜医务人员的事件，10人受伤，6人重伤。警方在案发现场抓获6名犯罪嫌疑人。 1月31日8时，患者刘某家属因一起医患纠纷，在该院急诊楼及门口设灵堂、拉横幅，严重影响正常医疗秩序和车辆通行。医院报警后，“110”及时出警，但劝说无果。 当天10时30分，患者刘某家属约20人，直接冲入该院心胸外科病区，踢开心胸外科主任办公室的门，发现室内无人，转而攻击隔壁办公室的心胸外科副主任，不容分说，凶手连续2刀捅向该医生的左前胸，致其当场重伤倒地。之后凶手还将其拖至八楼窗口，企图将他从窗口推下。其他医务人员见状，奋不顾身与凶手搏斗。在制止犯罪行为中，有10名医务人员受伤，其中6位医生伤情严重住院治疗，伤情最重者左前胸伤口深达4厘米，离心脏仅1.5厘米，造成血气胸，险些致命。受伤的6名男性医生均为年轻的医学博士、医疗骨干，并且都不是该患者的经治医生。 1月31日中午，警方在案发现场抓获6名犯罪嫌疑人。目前，已有1人被警方刑事拘留，3人被治安拘留，2人被治安警告。  A serious injury to innocent medical staff occurred in the Department of CARD Io thoracic Surgery of a Shanghai hospital, 10 people were injured, 6 seriously. The police arrested six suspects at the scene of the crime.  At 8 o 'clock on January 31, due to doctor-patient disputes，patient Liu's family set up memorial halls and hung banners in the hospital's emergency building and the main entrance. Such an act severely affected the normal medical treatment order and the flow of vehicles. The hospital called the police for help and they came out in time, but still failed to persuade the patient's family.  At 10:30 on the same day, the patient Liu's family of about 20 people, directly into the hospital cardiothoracic surgery ward, kicked open the door of the cardiothoracic surgery director office, found no one inside, turned to attack the cardiothoracic surgery deputy director who in the next office, allow no excuse, the murderer consecutive 2 knife stabbed to the doctor's left front chest, causing its serious injury on the spot fell to the ground. The killer then dragged him to an eighth-floor window and tried to push him out. Other medical staff saw this, desperate to fight with the killer. In the course of the crime, 10 medical personnel were injured, six of whom were hospitalized with serious injuries, the most serious of which was a wound of 4 cm deep in the left front chest, just 1.5 cm from the heart, resulting in a hemopneumothorax that was almost fatal.  The six male doctors who were injured are all young medical doctors and medical backbone, and none of them was the doctor who treated the patient. At noon on January 31, police arrested six suspects at the scene of the crime. At present, there have been 1 person by the police criminal detention, 3 people by the public order detention, 2 people by the public order warning. | | 318 | 1.19 | | .477 | | | 1.45 | | | | .768 | | 6.42 | 2.029 | | 4.87 | | 1.910 |
| 3 | | 2019年12月4日，95岁的孙某的母亲被送到某医院诊科，经诊断属于肿瘤晚期，且有脑梗塞后遗症等多种病症，属于典型“高龄慢性病人”。当天，负责首次接诊的是急诊科副主任医师杨医生。 按照正常流程，杨医生提出让孙某的母亲做相应检查。而家属拒绝就相关检查签字，仅要求输液。后期，则以急诊花销大为由，要求安排孙某母亲住院。一位医生回忆，因病情较重，当天本要由急诊科转入肿瘤科病房，但正值年底，住院部肿瘤科的重症监护室床位已满，只得留在急诊科的抢救室。 一位该医院的医生，谈到当时的情况：“家属签字拒绝一切检查，仅要求输点液，但是输液后病情无改善好转，几个家属就认定是杨医生输液给输坏了。” 之后的半个月内，据该医院医生回忆，一家人不接受疾病、不接受死亡，每天都会因为细微病情变化和怀疑医生用药，吵闹、辱骂、威胁。“我们建议病人转院，建议家属走医疗鉴定，都不同意。他们就在抢救室天天跟我们干架”。 “我们当时很明确讲了，住院床位紧张，建议他们转到其他医院。”民航总医院急诊科工作人员称，此后即发生争吵，医生报案两次，警方也进行了劝说。 悲剧发生在12月24日凌晨6点多。在杨医生伏案工作时，孙某和她交流了20分钟。突然从背后揪住她头发，扼住她颈部，连砍数刀。 一个小时后，抢救小组到达了该医院参与抢救。但长达13个小时的抢救没能挽回杨医生的生命。  On December 4, 2019, 95-year-old Sun's mother was admitted to the clinic of a hospital. She was diagnosed with advanced cancer、sequelae of brain infarction and multiple diseases ,as a typical "elderly chronic patient" .On the same day, responsible for the first clinical reception is the emergency department deputy chief physician Dr. Yang. In accordance with the normal process, Dr. Yang put forward to let Sun's mother do the corresponding examination. The family refused to sign for the tests and asked only for Infusion.  Later, on the grounds of high emergency expenses, the request to arrange Sun's mother in hospital. A doctor recalled that because of her serious condition, she was supposed to be transferred from the emergency department to the oncology ward that day, but at the end of the year, the intensive care unit of the inpatient oncology department was full, so she had to stay in the emergency room of the emergency department. A doctor in the hospital, talked about the situation at that time: "The family members signed to refuse all the examination, only asked to Infusion, but the condition did not improve after infusion, several family members affirmed that it was the transfusion made by Dr. Yang that led to a bad result." During the next half month, doctors at the hospital recalled that the family refused to accept illness or death. Every day, they quarreled, abused and threatened doctors because of subtle changes in medical condition of Sun's mother and suspicions about medication."  We advised the patient to be transferred to another hospital and the family to go for a medical evaluation, but they did not agree. They fought with us every day in the emergency room." "We made it very clear that there was a shortage of inpatient beds and we advised them to transfer to other hospitals." According to emergency department staff of the Civil Aviation General Hospital, a quarrel broke out after that, and doctors reported the incident twice and the police intervened. The tragedy happened just after 6 a.m. on December 24. When Doctor Yang works at his desk, Sun and she communicated for 20 minutes. Suddenly grabbed her by the hair from behind, grabbed her neck, and slashed her multiple times. An hour later, the rescue team arrived at the hospital. But 13 hours of efforts failed to save Dr. Yang's life. | | 386 | 1.23 | | .504 | | | 1.73 | | | | 1.015 | | 5.83 | 2.520 | | 3.43 | | 1.755 |
| 4 | | 10月18日当晚9时30分左右，两名黑衣男子扶着一名花衫男子冲进某医院急诊室，只见其中一名黑衣男子不由分说一拳朝徐医生的眼部抡去，一记重拳下他疼得立刻捂住了双眼，趴在桌上好半天也没能缓过神来，鲜血溅在了他的白大褂上，地上也有斑斑血迹。医护人员立刻上前阻止打人男子，他们到底和徐医生有什么仇什么怨呢？ 据徐医生回忆，花衬衫男子当晚酒后被玻璃划伤肘部，造成约两条肌腱断裂，护士已经用纱块和绷带帮他止了血。他告诉记者，当时患者家属要他马上为患者做缝合手术，考虑到患者已经喝酒，无法进行麻药处理，如强行麻药手术，会引起患者的呕吐，严重时甚至会窒息身亡。 “当时包扎完伤口后，护士已经和患者及其家属解释过不能立刻手术的原因。”徐医生说，但三人并不理解，依旧在医院大吵大闹了40多分钟，还出手伤人。当晚，他立即报了警并被送往医院治疗。 徐医生不仅眼部受伤严重，还感到头晕恶心，医院诊断单显示他已被打成轻微脑震荡。而打人的患者家属邱某，也在石岐区莲峰派出所受到了行政处罚，派出所提供的行政处罚决定书显示，邱某被处以行政拘留十日并罚款三百元。如今，邱某已经从拘留所出来恢复了自由，然而徐医生却说，至今他也没有听到来自对方的一句道歉。 徐医生的眼睛原本好好的，现在经过测试视力下降到只有0.4，需要戴上几百度的眼镜才能看清东西。据他透露，自己已经支付了近万元医疗费。对方不仅没有一点表示，令他感到心寒的是，连一句简单的“对不起”也没有听到。医生肩负着救死扶伤的重任，谁又来保护他们的基本权益不受侵犯呢？  At about 9:30 on the evening of October 18, two men in black rushed into the emergency room of a hospital with a man in a colored shirt. One of the men in black threw a punch at Dr. Xu's eyes. With a heavy punch, he immediately covered his eyes with pain. He lay on the table for a long time without calming down. Fresh blood splashed on his white coat and there were blood stains on the ground. The medical staff immediately went forward to stop the man from beating. What exactly did they hate Doctor Xu?  According to Dr. Xu, the man with flowery shirt was injured on his elbow by glass after drinking that night, causing about two tendons to break. The nurse has stopped his blood with gauze and bandages. He told the reporter that at that time, the patient's family asked him to perform suture operation for the patient immediately. Considering that the patient had already drunk, he could not perform anesthetic treatment. For example, forced anesthetic operation would cause the patient to vomit, and even suffocate to death in serious cases.  "After dressing the wound at that time, the nurse had explained to the patient and his family why they could not operate immediately." Doctor Xu said that the three people did not understand, but they still made a scene in the hospital for more than 40 minutes and hurt others. That night, he immediately called the police and was sent to the hospital for treatment.  Doctor Xu not only suffered serious eye injuries, but also felt dizzy and nauseated. The hospital diagnosis sheet showed that he had been beaten into a mild concussion. Qiu, the family member of the victim, also received an administrative penalty at the Lianfeng Police Station in Shiqi District. According to the written decision on administrative penalty provided by the police station, Qiu was detained for 10 days and fined 300 yuan. Now, Qiu has recovered his freedom from the detention center. However, Dr. Xu said that up to now, he has not heard an apology from the other side.  Doctor Xu's eyes were originally good, but now after testing, their vision has dropped to only 0.4, and they need to wear several Baidu glasses to see things clearly. According to him, he has paid nearly 10000 yuan for medical treatment. Not only did the other party not say anything, but to his horror, he did not even hear a simple "sorry". Doctors shoulder the heavy responsibility of saving the dying and healing the wounded. Who will protect their basic rights and interests from infringement? | | 436 | 1.30 | | .952 | | | 1.80 | | | | 1.324 | | 5.93 | 2.434 | | 4.50 | | 2.146 |
| 5 | | 11月10日下午4点20分左右，广东珠海一医院出现一起病患家属伤医事件。据悉，被打的为当日的值班护士，而事情缘由则是，护士依照当下防疫规定，拒绝了没有核酸检测报告的家属进入病房探视。记者获悉，当事护士目前颈部、手臂等处受伤，正在医院住院观察。依据香洲公安分局通报，目前嫌疑男子刘某已被行政拘留。 11日上午，记者在见到了正在神经外科住院观察的被打的左护士。谈起昨日的经历，惊魂未定的左护士当场掉下眼泪。“医护人员被打的这种事情，我以前只在电视上新闻上看过，没想到会发生在自己身上。”她说。 左护士描述，10日下午4点十多分的时候，在该医院肿瘤楼A座，一名男子准备进去病房，被正在值班的她便拦住询问。她表示，按照医院的规章制度，疫情期间要求原则上禁止探视，确因病情原因需要探视的，严格做好探视人员的流调筛查，以及要求提供近期核酸结果方可允许探视。几番沟通后，男子突然变得很激动，期间还一度掐住了她的脖子。左护士表示，自己当时被吓坏了，好在其他医护人员见状赶紧过来帮忙，阻止了男子下一步的行动。 据悉，在左护士遭遇暴力行为后，院方迅速报警。 记者看到，左护士昨天被掐脖子后的痕迹，依然十分清晰。她说，自己现在还是有点头晕，有时候会喘不过气来。  At about 4:20 p.m. on November 10, a hospital in Zhuhai, Guangdong, witnessed an incident in which a patient's family member was injured. It is reported that the one who was beaten was the nurse on duty on that day, but the reason was that the nurse refused to allow the family members without nucleic acid test report to enter the ward to visit according to the current epidemic prevention regulations. The reporter learned that the nurse in question was injured in the neck, arms and other places and was hospitalized for observation. According to the report of Xiangzhou Public Security Branch, Liu, a suspected man, has been administratively detained.On the morning of the 11th, the reporter saw the beaten left nurse who was being observed in the neurosurgery department. Talking about yesterday's experience, the left nurse who was still in shock shed tears on the spot. "I used to only watch the news on TV when medical staff were beaten, but I didn't expect it would happen to me." She said. Nurse Zuo described that at 4:10 pm on the 10th, a man was going to enter the ward in the tumor building A of the hospital, and she stopped him to ask. She said that, according to the rules and regulations of the hospital, visiting was prohibited in principle during the epidemic. If it was really necessary to visit because of illness, the flow screening of visitors should be done strictly, and the recent nucleic acid results should be provided before visiting. After several times of communication, the man suddenly became very excited, and at one time he held her by the neck. Nurse Zuo said that she was frightened at that time. Fortunately, other medical staff rushed to help and stopped the man from taking the next step. It is reported that after nurse Zuo suffered violence, the hospital quickly called the police. The reporter saw that the left nurse was still very clear after being pinched yesterday. She said that she was still a little dizzy and sometimes could not breathe. | | 346 | 1.39 | | .659 | | | 2.39 | | | | 1.676 | | 4.82 | 2.270 | | 4.45 | | 2.237 |
| 6 | | 9月14日，产妇孙某（女，44岁）入院后完善相关检查，考虑诊断为慢性高血压合并妊娠。经产科主任查房讨论，考虑患者血压控制平稳，曾有阴道分娩史，无明确剖宫产指征，可行产房引产。这期间患者病情及情绪平稳，未提出任何诉求。 9月22日晚，值班一线张医生去产一病房查看病人时，在无任何防备下，被孙某丈夫郑某径直冲上来推搡，张医生受威胁后被迫返回产房。 当晚九时许，值班二线赫医生在做完手术后，在返回五楼产房途中，遇见患者丈夫郑某。产妇丈夫情绪激动，说话带着脏字质问道：“我爱人能生吗？”赫医生回答：“这个孕妇的情况，已经经过主任查房，目前可以经阴道试产”。谁知话音刚落，郑某情绪激动并突然动手殴打赫医生，医生躲避几下后，两人扭打在一起，随后被工作人员拉开，郑某仍不依不饶追了上去。身穿绿色工作服的赫医生被三名男女按着头部殴打，被旁边几名医生拉开后，又有人上前对着赫医生脸部挥拳，用脚踹其身上，直至医生趴在地上。其间，一位孕妇一直用手揪着该医生衣服。 据网友爆料，一位产妇不想自己生，但没有剖指征，家属就把医生打了。被打医生系医院妇产科医生；还有网友指认，视频中一名打人女子疑为某大学在读学生。  On September 14, Sun (female, 44 years old), a lying in woman, completed relevant examinations after admission and considered the diagnosis of chronic hypertension with pregnancy. After ward round discussion by the obstetric director, considering that the patient's blood pressure was controlled stably, had a history of vaginal delivery, and had no clear indication of cesarean section, it was feasible to induce labor in the delivery room. During this period, the patient's condition and mood were stable, and no appeal was made. On the evening of September 22, Dr. Zhang, the front-line doctor on duty, went to the maternity ward 1 to check the patient. Without any precaution, Dr. Zhang was rushed up and pushed by Mr. Sun's husband, Mr. Zheng. After being threatened, Dr. Zhang was forced to return to the delivery room. At about 9:00 that night, after the operation, Dr. He, the second line on duty, met the patient's husband, Mr. Zheng, on his way back to the delivery room on the fifth floor. The husband of the lying in woman was excited and asked with dirty words: "Can my wife give birth?" Dr. He replied: "The situation of the pregnant woman has been checked by the director, and now she can have a trial delivery via vagina.". Unexpectedly, just after his words ended, Mr. Zheng was excited and suddenly began to beat Dr. He. After the doctor dodged for a few times, they scuffled together, and then were pulled away by the staff. Mr. Zheng still chased him. Doctor He in green overalls was beaten on the head by three men and women. After being pulled away by several doctors nearby, another person came forward and punched Doctor He in the face and kicked him with his foot until the doctor lay on the ground. Meanwhile, a pregnant woman has been holding the doctor's clothes with her hands. According to netizens, a pregnant woman did not want to give birth by herself, but there was no Indications for Caesarean section, so her family beat the doctor. The beaten doctor is the obstetrician and gynecologist of the hospital; Some netizens pointed out that a woman in the video suspected that she was a student in a university. | | 370 | 1.48 | | .755 | | | 1.91 | | | | 1.234 | | 5.21 | 2.288 | | 4.55 | | 2.451 |
| 7 | | 今天下午16时，哈尔滨一医院发生一起恶性伤害案件，致一人死亡，三人受重伤。据目击者介绍，行凶者是一名约20岁的男子，现已被抓获，相关情况警方正在调查之中。 16时30分左右，该院风湿免疫科医务人员正在紧张地忙碌着，这时，一名男子突然闯入医生办公室，抡起手中的刀，疯狂砍向正在埋头工作的医务人员和实习学生，大家躲避不及，一名实习学生颈部鲜血喷涌。 医院迅速成立抢救小组，组织专家进行抢救。一名实习学生伤势严重不幸死亡，另三名医务人员重伤仍在抢救之中。死者为王医生，男，今年28岁，为09级硕士研究生，出事前刚刚收到香港中文大学博士录取通知书。凶手现已被警方控制，据悉，凶手约20岁，一年前曾在医院就诊，此次在其祖父陪同下来院咨询。其祖父在与医生详细咨询时，凶手一直在诊室外等候。在没有任何征兆情况下，凶手突然闯入另外一间医生办公室，向正在埋头工作毫无戒备的医生举刀就砍，行凶后欲自杀未果。 事件发生后，医院立即情况上报省卫生厅、教育厅及相关部门，卫生厅、教育厅和学校领导立即赶往该院，组成工作小组研究伤员救治及善后处理工作，医院已组织专人做好在场医生和学生的心理辅导，恢复医院秩序。  At 16:00 this afternoon, a malignant injury case occurred in a hospital in Harbin, causing one death and three serious injuries. According to the witness, the perpetrator was a man about 20 years old and has been arrested. The police are investigating the situation.  At about 16:30, the medical staff of the Rheumatology and Immunization Department of the hospital were busy nervously. At this time, a man suddenly broke into the doctor's office, swung his knife, and cut at the medical staff and interns who were working hard. Everyone could not escape. A student intern's neck was gushing blood. The hospital quickly set up a rescue team to organize experts for rescue. One trainee died of serious injuries, and the other three medical staff are still under rescue. The deceased was Doctor Wang, a 28 year old male graduate student of Grade 09. He had just received the admission notice of the Chinese University of Hong Kong before the accident. The murderer is now under the control of the police. It is reported that the murderer is about 20 years old. He was hospitalized a year ago. This time, he was accompanied by his grandfather to the hospital for consultation. When his grandfather was consulting with the doctor in detail, the murderer had been waiting outside the consulting room. Without any sign, the murderer suddenly broke into another doctor's office, raised his knife to the doctor who was working without warning, and tried to kill himself after the murder. After the incident, the hospital immediately reported the situation to the Provincial Department of Health, the Department of Education and relevant departments. The leaders of the Department of Health, the Department of Education and the school immediately rushed to the hospital, formed a working group to study the treatment of the wounded and the aftermath. The hospital has organized special personnel to provide psychological guidance to the doctors and students on the scene, and restore the hospital order。 | | 328 | 1.52 | | 1.004 | | | 2.12 | | | | 1.474 | | 5.61 | 2.657 | | 4.30 | | 2.352 |
| 8 | | 2018年12月14日上午，某医院发生一起伤医事件。嫌疑人曾某因医疗纠纷持刀将张医生捅伤后跳楼自杀身亡，受伤的医生生命垂危，医院正全力组织抢救。目前，警方已介入调查。 当日中午， 该医院通过官方微博发布称，医院一外科医生在门诊正常坐诊期间，被一身份不明人员持刀刺伤，生命垂危，医院目前正全力组织抢救。希望受伤医生能够尽快脱离生命危险。 ” 该医院工作人员告诉记者，该事件发生于14日上午，事件中有一名医生受伤，该名医生伤情很严重，医院一直在抢救。 该名工作人员表示，院方已经报警，警方也介入了该事件的调查，但因为该事件的调查结果还不是很清楚，具体情况需要晚一点才能公布。“发生这么大一件事情，我们需要配合公安部门调查，这个事情可能会指向医患关系。”该名工作人员对记者说。 14日下午1时，该地区公安分局官方微博发布称，今天上午11时许，嫌疑人曾某（男，51岁，患者）在医院因医疗纠纷持刀将医生张医生（男，49岁）捅伤后跳楼自杀身亡。目前，正在全力抢救张医生中，案件原因调查及相关工作正在进行中。  On the morning of December 14, 2018, a medical accident occurred in a hospital. The suspect Zeng, who stabbed Doctor Zhang with a knife due to a medical dispute, jumped off a building and committed suicide. The injured doctor's life was in critical condition, and the hospital was making every effort to organize rescue. At present, the police have been involved in the investigation. At noon on the same day, the hospital announced on its official microblog that a surgeon in the hospital was stabbed by an unidentified person with a knife during a normal outpatient clinic, and his life was in critical condition. The hospital is now making every effort to organize rescue, and hope the injured doctor can get out of danger as soon as possible. " The hospital staff told reporters that the incident occurred on the morning of the 14th, and one doctor was injured in the incident. The doctor was seriously injured and the hospital has been rescuing him.  The staff member said that the hospital had called the police and the police had also been involved in the investigation of the incident, but because the investigation results of the incident were not very clear, the specific situation would need to be announced later. "We need to cooperate with the public security department to investigate such a big thing, which may point to the doctor-patient relationship," the staff member told reporters. At 1 p.m. on the 14th, the official microblog of the local public security bureau said that at about 11 a.m. this morning, the suspect Zeng (male, 51 years old, patient) jumped from a building to commit suicide after stabbing doctor Zhang (male, 49 years old) with a knife in the hospital due to a medical dispute. At present, we are trying our best to rescue Doctor Zhang, and the investigation of the cause of the case and related work are in progress. | | 318 | 1.53 | | .900 | | | 2.00 | | | | 1.259 | | 5.50 | 2.515 | | 3.63 | | 1.732 |
| 9 | | 据甘肃省兰州市公安局城关公安分局官方微博消息，10月22日上午，犯罪嫌疑人杨某（男，54岁，兰州市人）在甘肃省人民医院持刀对医生冯某某（女，42岁，兰州市人）行凶，虽经全力抢救，冯某某不幸离世。 事发时犯罪嫌疑人杨某像往常一样前往受害者所在的肛肠科，途中并未有任何出格举动和异常。因为杨某身患直肠癌，所以身为肛肠科内主治医师的冯某便是杨某的寻找对象。 而一直以来，冯某也作为杨某的主治医师负责杨某住院期间的一切咨询、治疗、恢复。案发时，54岁的杨某突然挥刀刺向自己的主治医生冯某！致使冯某闪躲不及，被刺重伤，抢救无效死亡，而冯医生年仅42岁，家中还有两个孩子。 凶手杨某案发后随即被公安机关控制。经初步调查，杨某因患直肠癌，曾在省人民医院就诊手术，医生冯某某为其主治医师。目前案件正在进一步侦办中。 据了解，被害女医生正是该院的肛肠科医生冯医生。据知情者说，杨某是在该院门诊大楼三楼袭击冯医生的，当时就冯医生医生一人在，凶手还在现场释放了刺激性不明液体，现场有浓烟冒出。据说，冯医生刚下乡回来。  According to the official microblog of Chengguan Public Security Branch of Lanzhou Public Security Bureau in Gansu Province, on the morning of October 22, the suspect Yang (male, 54 years old, from Lanzhou City) attacked the doctor Feng (female, 42 years old, from Lanzhou City) with a knife in the People's Hospital of Gansu Province. Despite all efforts to rescue him, Feng died unfortunately. At the time of the incident, the suspect, Mr. Yang, went to the anorectal department where the victim was, as usual, without any unusual behavior or abnormality on the way. Because Yang suffers from rectal cancer, Feng, who is the chief physician in the anorectal department, is the target of Yang. For a long time, Feng, as the chief physician of Yang, was also responsible for all the consultations, treatment and recovery of Yang during his hospitalization. At the time of the crime, 54 year old Yang suddenly stabbed his doctor Feng! As a result, Feng could not dodge, was seriously injured, and died of ineffective rescue. However, Doctor Feng was only 42 years old and had two children at home. The murderer Yang was immediately controlled by the public security organ after the crime.  According to the preliminary investigation, Yang had seen an operation in the Provincial People's Hospital due to rectal cancer, and Feng was the doctor in charge. At present, the case is under further investigation. It is understood that the murdered female doctor is Dr. Feng, the anorectal doctor of the hospital. According to insiders, Yang attacked Dr. Feng on the third floor of the outpatient building of the hospital. At that time, Dr. Feng was alone. The murderer also released an unknown irritant liquid at the scene, and thick smoke was emitted at the scene. It is said that Doctor Feng has just returned from the countryside. | | 306 | 1.57 | | 1.547 | | | 1.93 | | | | 1.530 | | 5.63 | 2.632 | | 4.30 | | 1.896 |
| 10 | | 因在新冠肺炎疫情期间强行闯入医院，辱骂、殴打医生和保安，温州龙港市两名男子日前被法院以寻衅滋事罪分别判处有期徒刑。  4月19日凌晨，陈某在外喝酒时得知女友因烫伤在龙港医院治疗，便前往医院。此时正值疫情防控关键期，陈某到医院门口后却不服从管理，故意不走人行通道，徒手推断停车道闸杆后闯入。 在急诊室，他干扰医生钟某对伤情的诊治，又认为其态度生硬，对其进行辱骂并形成肢体冲突。医生胡某上前劝阻，遭到陈某殴打。保安秦某等赶来将陈某制服，但陈某挣脱，继续对钟某拳打脚踢。陈某的朋友林某得到陈某在医院被“欺负”的消息，手持不锈钢水瓢赶到现场，伙同陈某殴打保安秦某等人。后二人被赶来的民警带走。 经鉴定，胡某的损伤程度为轻伤二级，钟某、秦某为轻微伤。 龙港法院审理认为，被告人陈某随意殴打他人、情节恶劣，被告人林某持凶器随意殴打他人，均构成寻衅滋事罪，判处陈某有期徒刑三年四个月、林某有期徒刑一年十个月。  During the COVID-19, two men in Longgang, Wenzhou, were sentenced to fixed-term imprisonment for provocation and trouble making by the court for forcibly entering the hospital, abusing and beating doctors and security guards.  In the early morning of April 19, when Chen was drinking outside, he learned that his girlfriend was being treated in Longgang Hospital due to scald, so he went to the hospital. At this time, it was the critical period for epidemic prevention and control. After arriving at the hospital gate, Mr. Chen did not obey the management, deliberately did not walk through the pedestrian passage, and broke in after inferring the brake lever of the parking lane with his bare hands.  In the emergency room, he interfered with the doctor Zhong's diagnosis and treatment of the injury, and thought that his attitude was stiff, so he abused him and formed a physical conflict. Hu, the doctor, came forward to dissuade him and was beaten by Chen. Qin and other security guards rushed to subdue Chen, but Chen broke away and continued to beat and kick Zhong. Lin, a friend of Chen, got the news that Chen was "bullied" in the hospital, and rushed to the scene with a stainless steel water ladle, helping Chen beat the security guard Qin and others. The latter two were taken away by the police.  After identification, Hu's injury was classified as minor injury, while Zhong and Qin's injury was classified as minor injury.  The Longgang Court held that the defendant Chen beat others at will and the circumstances were abominable, and the defendant Lin beat others at will with a murder weapon, which constituted the crime of provoking trouble and sentenced Chen to three years and four months in prison and Lin to one year and ten months in prison. | | 300 | 1.61 | | .715 | | | 2.29 | | | | 1.243 | | 5.58 | 2.306 | | 4.77 | | 1.586 |
| 11 | | 4月29日凌晨3时19分，曲靖市内某地发生车祸，120急救中心到达现场检查发现男性伤者已经死亡。随后被救出的女性伤者经急救后于4时40分送至曲靖市某医院，经手术抢救无效后于12时40分死亡。 之前，男性死者张某于凌晨4时4分由交警通知医院太平间管理人员进行处理，由于市一院太平间已满，经联系，于凌晨4时40分左右将尸体送入市二院太平间。凌晨5时30分左右，太平间接到家属电话要求看死者尸体，而7时10分左右，家属突然说死者还有体温，并认为还有心跳，便强行将尸体推送到市二院急诊科。 医务人员在对当夜情况一无所知的情况下，按急诊抢救程序进行了抢救，而经医生临床诊断和7时18分打印心电图证实，伤者无生命体征，而家属仍要求继续抢救。 7时26分，医务人员第二次打印心电图，告诉家属伤者无生命体征，家属随即情绪失控开始动手打医生护士，保卫人员到达后与家属发生了肢体接触。在整个过程中，家属仍要求医生在家属同意前不能停止抢救，抢救操作持续到8时50分。 据了解，市二院2名值班的医生和护士被打伤，住院治疗；1名副院长被打伤，门诊观察治疗，4名保卫人员被打伤，门诊观察治疗。经曲靖市卫生局、麒麟区公安局现场做工作，死者家属当日14时20分许将尸体再次移送市二院太平间保存。 事件发生后，曲靖市卫生局长及时赶到现场，抽调人员组成三个调查组查明：市一院、市二院在对本次交通事故伤员的接诊抢救治疗过程中，未有违反医疗原则和诊疗规范行为。同时也未发现医务人员渎职、失职、推诿伤者的现象。事实证明，市二院医务工作者纯属无故被打伤。  At 3:19 a.m. on April 29, a car accident occurred in a place in Qujing City. The 120 Emergency Center arrived at the scene and found that the male wounded had died. Later, the rescued female wounded were sent to a hospital in Qujing City at 4:40 after first aid, and died at 12:40 after surgery.  Previously, Zhang Mou, the male victim, was informed by the traffic police to the hospital mortuary management personnel at 4:4 a.m. for disposal. As the mortuary of the First Municipal Hospital was full, the body was sent to the mortuary of the Second Municipal Hospital at about 4:40 a.m. after contact. At about 5.30 a.m., the mortuary received a call from the family members asking to see the dead body. At about 7.10 a.m., the family members suddenly said that the dead body still had temperature and thought that it still had heartbeat, so they forced the body to the emergency department of the Second Municipal Hospital.  The medical staff carried out the rescue according to the emergency rescue procedure when they knew nothing about the situation that night. However, the doctor's clinical diagnosis and the 7:18 printed ECG confirmed that the injured person had no vital signs, while the family members still requested to continue the rescue.  At 7:26, the medical staff printed the ECG for the second time and told the family members that the injured had no vital signs. The family members immediately lost control of their emotions and began to beat the doctors and nurses. After the security staff arrived, they had physical contact with the family members. During the whole process, the family members still asked the doctor not to stop the rescue until the family members agreed, and the rescue operation continued until 8:50.  It is understood that two doctors and nurses on duty in the Second Municipal Hospital were injured and hospitalized; One vice president was injured and observed and treated in the outpatient department, and four security personnel were injured and observed and treated in the outpatient department. After Qujing Municipal Health Bureau and Qilin District Public Security Bureau did on-site work, the family members of the deceased again transferred the body to the mortuary of the Second Municipal Hospital for preservation at about 14:20 on the same day.After the incident, Qujing Municipal Health Director rushed to the scene in time and transferred personnel to form three investigation teams to find out that the First and Second Municipal Hospitals did not violate the medical principles and diagnostic and therapeutic norms in the process of receiving, rescuing and treating the injured in the traffic accident. At the same time, no malpractice, dereliction of duty and evading the injured were found. Facts have proved that the medical workers of the Second Municipal Hospital were injured for no reason. | | 471 | 1.70 | | 1.317 | | | 1.63 | | | | .890 | | 5.53 | 2.583 | | 3.43 | | 1.382 |
| 12 | 四川泸州12岁的男孩小鑫曾因重病入院，与死神擦肩而过，在医护人员的全力救治下，终于转危为安。在病情逐渐好转后，从未学过绘画的小鑫拿起画笔，为帮助过他的每一名医护人员画了一幅铅笔画，表达敬意和感谢。 今年9月初，小鑫因病住进了西南医科大学附属医院康健中心院区的儿童重症监护室（PICU）病房，当时他有高热、意识不清、凝血功能障碍等情况，病情十分危重。 当时小鑫还不知道的是：沈教授、邓医生、庄医生等人在他病床前轮流守护了72小时，直到他开始排尿、有自主呼吸后，才松了一口气，随后团队全力救治，终于把他从死亡线上拉了回来。 在PICU住了10天后，小鑫转回小儿外科病房，由护士继续予护理。经过两个多月的治疗，小鑫的病情逐渐好转。 在得知了医生、护士的帮助后，小鑫决定，画一些画送给他们，感谢他们的救命之恩。坐在病床上，小鑫拿起铅笔、撑起小手指，在白纸上描绘线条，不多久，一个米老鼠就跃然纸上。 小鑫住院期间，他与小儿外科的医护人员也渐渐熟悉起来，送给他们很多铅笔画。小鑫出院前，医护人员也准备了自己的小礼物，把美好祝福回馈给孩子。 小儿外科李护士说，一份份的小礼物，都满载着医护人员对小鑫的祝福，希望他早日康复，继续画下去。  Xiao Xin, a 12-year-old boy from Luzhou, Sichuan Province, was once admitted to the hospital due to a serious illness and passed by the god of death. With the full treatment of medical staff, he finally turned the corner. After his illness gradually improved, Xiaoxin, who had never learned painting, picked up a brush and drew a pencil drawing for each of his medical staff to express his respect and gratitude.  At the beginning of September this year, Xiaoxin was admitted to the Children's Intensive Care Unit (PICU) of the Health Center of the Affiliated Hospital of Southwest Medical University due to illness. At that time, he had high fever, confusion, coagulation dysfunction and other conditions, and his condition was very critical.  What Xiaoxin didn't know at that time was that Professor Shen, Dr. Deng, Dr. Zhuang and others took turns to guard in front of his bed for 72 hours. They were relieved when he started urinating and had spontaneous breathing. Then the team made every effort to cure him and finally pulled him back from the death line.  After staying in PICU for 10 days, Xiaoxin returned to the pediatric surgery ward, where the nurse continued to provide nursing care. After more than two months of treatment, Xiaoxin's condition gradually improved.  After learning about the help of doctors and nurses, Xiaoxin decided to draw some pictures for them to thank them for saving their lives. Sitting on the bed, Xiaoxin picked up a pencil, held up his little finger, and drew lines on the white paper. Soon, a Mickey Mouse appeared on the paper.  During Xiaoxin's hospitalization, he became familiar with the medical staff of pediatric surgery and gave them many pencil drawings. Before Xiaoxin left the hospital, the medical staff also prepared their own small gifts to give back their good wishes to the children.  Nurse Li, Pediatric Surgery Department, said that each small gift was filled with the blessings of the medical staff for Xiao Xin, hoping that he would recover soon and continue painting. | | 339 | | | | | 1.70 | | 1.317 | | 1.63 | | .890 | | 5.53 | 2.583 | | 3.43 | | 1.382 |
| 13 | 10月27日下午5时许，南昌市第一医院发生一起女护士被不明男子持刀挟持事件，约一小时后被劫持的女护士获救，所幸只是颈部和手腕受到了一点轻伤 　　“他拿着一把长约10厘米的水果刀，抵住我的脖子问我要电话，说是要报警。”刘护士说，当时她的电话并未带在身上。这个时候，在观察室内工作的万护士看见了，便询问男子需要什么帮助，没想到男子放掉刘护士，把万护士拖到了抢救室，关上大门不再出来。 　　昨日傍晚6时30分许，记者见到了惊魂未定的万护士。万护士说，在被劫持进抢救室后，男子情绪十分激动，问她要手机。她一边说话帮男子放松，一边将手机递给了男子，但男子无法打开手机，情绪更加激动，并将手机丢掉。“他说他今年23岁，劫持我是因为家庭纠纷。当时外面有人说话，谈到了‘家’这个字。他大概以为不会有人帮他，便咬牙切齿，情绪十分激动，并一直说要见妈妈。” 万护士回忆，在被劫持的近一小时内，男子拿着水果刀在她脖子上来回比划，划伤了她的脖子 “我感觉他情绪很激动，要用刀刺我，就趁他不注意的时候，使出浑身力气把水果刀打掉在地上，然后大喊了一声。”守候在门口的民警听到叫声立刻冲进去，控制住了劫持者。据了解，挟持事件发生后，东湖公安分局主要领导率刑侦大队、百花洲派出所所有民警赶到了现场，并请求南昌市特警支队增援。 “被劫持的护士小万的颈部伤口尽管不深，但离颈动脉只差了一点点，非常危险。此外，其手腕在打落歹徒凶器时受了一点轻伤，因惊吓等原因，精神受到了很大刺激，目前需要进一步观察治疗。晚上6时30分许，嫌疑男子被民警带离医院。  At about 5:00 p.m. on October 27, a female nurse in Nanchang First Hospital was kidnapped by an unknown man with a knife. About an hour later, the kidnapped female nurse was rescued. Fortunately, she suffered minor injuries to her neck and wrist.  "He held a 10 cm long fruit knife against my neck and asked me to call, saying that he would call the police." Nurse Liu said that her phone was not with her at that time. At this time, nurse Wan, who was working in the observation room, saw it and asked the man what help he needed. Unexpectedly, the man let nurse Liu go and dragged nurse Wan to the rescue room, closing the door and never coming out again.  At about 6:30 yesterday evening, the reporter saw the frightened nurse Wan. Nurse Wan said that after being kidnapped into the rescue room, the man was very excited and asked her for her mobile phone. While talking, she helped the man relax and handed him the mobile phone, but the man could not open it, so he became more excited and threw it away. "He said he was 23 years old and kidnapped me because of a family dispute. At that time, someone outside talked about the word 'home'. He probably thought that no one would help him, so he gritted his teeth and was very excited. He always said he wanted to see his mother."  Nurse Wan recalled that within nearly an hour of being kidnapped, the man held a fruit knife and stroked back and forth on her neck, cutting her neck. "I felt that he was very excited and wanted to stab me with a knife. When he was not paying attention, I used all my strength to knock the fruit knife off the ground, and then shouted." The police waiting at the door rushed in immediately and controlled the hijackers. It is understood that after the hijacking incident, the main leaders of the East Lake Public Security Branch led the criminal investigation team and all the police of the Baihuazhou Police Station to the scene and requested the Nanchang Special Police Detachment to reinforce.  "Although the neck wound of the kidnapped nurse Xiao Wan was not deep, it was only a little bit from the carotid artery, which was very dangerous. In addition, her wrist was slightly injured when she knocked down the gangster's weapon. Because of fright and other reasons, her spirit was greatly frightened, and now she needs further observation and treatment. At about 6:30 p.m., the suspect man was taken out of the hospital by the police. | | 438 | | | | | 1.76 | | .969 | | 2.27 | | 1.398 | | 4.91 | 2.416 | | 4.27 | | 2.035 |
| 14 | 6月18日8：00，南平市延平区太平镇杨厝村村民杨某，因“泌尿系结石、急性肾功能衰竭”入住南平市第一医院泌尿外科。医生在与患者家属充分沟通告知术中术后风险，患者家属在手术知情同意书上签字后，于6月20日对患者施行手术，手术顺利。 6月20日22：10，患者突发变症，经抢救无效于6月21日0：20宣布死亡。对此，死者家属认为院方对患者的死亡负有责任，当晚就聚集在泌尿外科，要求院方做出解释，拒不移尸，同时将参与抢救的泌尿外科主任胡医生和王医生扣留在病房，并提出高额赔偿要求。 6月21日7：30，死者家属到医院门诊大楼打横幅、摆花圈、烧纸钱，封堵大门通道，并将4名医务人员滞留在死者病房内。6月21日起，南平市、延平区两级政府和卫生、公安部门及患者所在镇、村领导多次协调处理，均因双方在赔偿金额上分歧较大，协商未果，并发生肢体冲突，双方均有人员受伤。后经多方做工作，死者家属与医院达成初步协议。  At 8:00 on June 18, Yang, a villager from Yangcuo Village, Taiping Town, Yanping District, Nanping City, was admitted to the Department of Urology of the First Hospital of Nanping City due to "urolithiasis and acute renal failure". The doctor fully communicated with the patient's family members to inform them of the risks during and after the operation. After the patient's family members signed the informed consent form for the operation, they performed the operation on the patient on June 20, and the operation was successful.  At 22:10 on June 20, the patient suddenly became ill and was declared dead at 0:20 on June 21 after rescue. In this regard, the family members of the deceased believed that the hospital was responsible for the death of the patient. They gathered in the urology department that night and asked the hospital to explain and refuse to remove the body. At the same time, they detained Dr. Hu and Dr. Wang, the head of the urology department who participated in the rescue, in the ward and asked for high compensation.  At 7:30 on June 21, the family members of the deceased went to the outpatient building of the hospital to put up banners, place wreaths, burn paper money, block the gate passage, and detain four medical staff in the ward of the deceased. Since June 21, Nanping City and Yanping District governments, health and public security departments, as well as the leaders of the town and village where the patient lives, have coordinated and handled for many times. Both parties failed to reach an agreement on the amount of compensation, and physical conflicts occurred, causing injuries to both parties. After many efforts, the family members of the deceased reached a preliminary agreement with the hospital. | | 293 | | | | | 2.06 | | 1.321 | | 2.55 | | 1.481 | | 4.91 | 2.416 | | 4.76 | | 2.208 |
| 15 | 据福建省卫计委通报，6月5日上午，福建省一医院发生一起严重暴力伤医事件。在专家门诊坐诊的省立医院耳鼻咽喉科行政主任、李医生被人袭击。 暴力伤医事件发生后，福建省立医院立即组织抢救，术中发现李医生全身多处刀伤(包括颈部一道刀痕)，右手拇长伸肌腱断裂，左第2—4指腱鞘损伤，左食指固有神经断裂。 该医院透露，手术持续3个多小时，出血量300-400ml。目前，李医生病情稳定。 伤医事件发生后，福建省卫生计生委高度重视，立即到省立医院了解情况，代表省卫计委对李医生表示慰问，强烈谴责暴力伤医行为，要求医院做好安全保卫工作，切实维护医院正常工作秩序，保障医务人员的人身安全。  According to the report of the Health and Family Planning Commission of Fujian Province, on the morning of June 5, a hospital in Fujian Province suffered a serious violent medical injury. Doctor Li, the administrative director of the Department of Otolaryngology of the provincial hospital in the expert clinic, was attacked.  After the medical violence incident, Fujian Provincial Hospital immediately organized rescue. During the operation, Dr. Li was found to have multiple stab wounds (including a knife mark on the neck), tendon rupture of the extensor hallucis longus of the right hand, tendon sheath injury of the left 2-4 fingers, and rupture of the proper nerve of the left index finger.  The hospital revealed that the operation lasted more than 3 hours and the amount of bleeding was 300-400ml. At present, Dr. Li's condition is stable.  After the medical injury incident, the Fujian Health and Family Planning Commission attached great importance to it and immediately went to the provincial hospital to learn about the situation. On behalf of the provincial Health and Family Planning Commission, it expressed condolences to Dr. Li, strongly condemned the violent medical injury, required the hospital to do a good job in security, effectively maintain the normal working order of the hospital and ensure the personal safety of medical personnel. It is understood that suspect Lin, a male, 84 years old, from Minhou County, Fuzhou City, once performed "right vocal cord polypectomy" in the hospital in March 2009 due to "right vocal cord polyp". After operation, he was dissatisfied with the therapeutic effect and complained many times. After the technical appraisal of Fuzhou medical accident, it was not a medical accident. | | 274 | | | | | 2.12 | | 1.474 | | 2.212 | | 1.409 | | 4.79 | 2.382 | | 4.03 | | 2.054 |
| 16 | 19日凌晨，内蒙古包头市一医院的朱医生在出诊时，被患者的丈夫用菜刀砍死。目前，犯罪嫌疑人已被刑事拘留。昨天，包钢医院官方网站首页，挂出被害女医生——朱医生的照片，两边均有蜡烛以示哀悼。 　　据医院官网介绍，当天凌晨1时42分，该医院急诊科接到包头市120指挥中心急救出诊电话：“在包头市昆都仑区友谊19街坊五区22栋90号，有一名女性患者突发抽搐，指派包钢医院出车进行急救。”随后，值班医生神经内科主治医师朱医生带领120急救队，立即出诊到达现场。“朱医生手提急救箱，第一时间冲上六楼，1名护士、2名担架工紧随其后，敲开房门后，患者家属称发病患者在阳台，朱医生立即直奔阳台，患者家属手持菜刀将担架工及护士砍伤并拦截在门外，随后把家门反锁，将朱医生杀害。” 　　昨晚，包头市公安局官方微博“平安包头”发文称：19日凌晨1时58分，包头市公安局110指挥中心接到报警：昆都仑区友谊19街坊五区22栋90号发生杀人案。接警后，公安民警迅速赶到现场并控制了犯罪嫌疑人。  In the early morning of the 19th, Dr. Zhu from a hospital in Baotou, Inner Mongolia, was killed by the patient's husband with a kitchen knife when he was on a call. At present, the suspect has been held in criminal detention. Yesterday, on the home page of the official website of Baogang Hospital, a photo of the murdered female doctor, Zhu, was displayed, with candles on both sides as a sign of mourning.  According to the official website of the hospital, at 1:42 am that day, the emergency department of the hospital received an emergency call from Baotou 120 Command Center: "At No. 90, Building 22, Block 5, Youyi 19, Kundulun District, Baotou City, a female patient suddenly twitched and assigned Baogang Hospital to drive out for emergency treatment."  Subsequently, the duty doctor, Dr. Zhu, the chief physician of neurology, led the 120 emergency team to visit the site immediately. "Doctor Zhu rushed to the sixth floor with the first-aid kit in his hand. One nurse and two stretchers followed him. After knocking on the door, the patient's family said that the patient was on the balcony. Doctor Zhu immediately went to the balcony. The patient's family held a kitchen knife to cut the stretcher worker and the nurse and stopped them outside the door. Then they locked the door and killed Doctor Zhu."  Last night, the official microblog of Baotou Public Security Bureau, "Ping An Baotou", reported that at 1:58 a.m. on the 19th, the 110 Command Center of Baotou Public Security Bureau received an alarm that there was a murder at No. 90, Building 22, Block 5, Youyi 19, Kundulun District. After answer the alarm, the public security police rushed to the scene and controlled the suspect.  It was found that on the evening of January 18, the suspect, Mr. Li, had a quarrel with his wife because of online shopping. After his wife fell asleep, he beat her up and injured her. His son sent his mother to the hospital for treatment. At 1:42 on the 19th, the suspect called 120 for help, and four medical staff arrived at the scene in time. The suspect wounded two people with a kitchen knife, and locked the door of the room to kill the visiting doctor Zhu. At present, the suspect has been held in criminal detention and the case is under further trial. | | 396 | | | | | 2.19 | | 2.212 | | 1.77 | | 1.454 | | 6.32 | 2.072 | | 4.35 | | 1.942 |
| 17 | 今年55岁的廖先生，此前一直从事高空作业，经常在空中一呆就是好几个小时，然后也没时间去上厕所，憋尿都憋成了习惯。后面随着年龄的增长，前列腺开始慢慢出现问题。 “开始的时候我觉得没得啥子，哪个晓得后来就老火了，排尿都感觉困难，有些时候尿里还有血。”廖延波告诉记者，发现自己前列腺有问题后，他开始四处求医治疗，但尝试了很多办法，效果都不明显。后来，朋友给他推荐了德阳新华西医院。 据了解，廖延波到该医院后，医院的专家热情地接待了老廖，并对他的身体展开了全面检查。“医生告诉我我这个情况是由于长期憋尿引发前列腺炎，因为不重视导致了前列腺肥大。”廖延波说，在医生的建议下，他在医院进行了手术。 “手术后一个疗程我就感觉身体慢慢好转了，住院一个星期以后身体机能已恢复得差不多了。”廖延波表示，在住院期间，医院的医生护  士对他非常关心，照顾的比较细致，医生经常都来询问病情，这让他十分感动，于是决定给医院医生护士送一面锦旗致谢。  Mr. Liao, 55 years old, has been engaged in aerial work before. He often stays in the air for several hours, and then has no time to go to the toilet. He has become a habit of holding his urine. Later, with the growth of age, prostate problems began to appear slowly."At the beginning, it was nothing wrong, but later I found something had changed. It was difficult to urinate, and sometimes there was blood in the urine." Liao Yanbo told reporters that after he found that he had a problem with his prostate, he began to seek medical treatment everywhere. However, after trying a lot of methods, there is still no effect. Later, a friend recommended Deyang New Huaxi Hospital to him.  After Liao Yanbo arrived at the hospital, the experts warmly received Lao Liao and carried out a comprehensive examination of his body. "The doctor told me that this condition was due to prostatitis caused by long-term urine holding, which led to hypertrophy of the prostate due to neglect." Liao Yanbo said that he had an operation in the hospital with the doctor's advice.  "After a course of treatment after the operation, I felt my body was getting better gradually. After a week in hospital, my body functions had almost recovered." Liao Yanbo said that during his stay in hospital, the doctors and nurses in the hospital were very concerned about him and took good care of him. The doctors often came to ask about his illness, which made him very moved. So he decided to send a banner to the doctors and nurses in the hospital to thank him. | | 272 | | | | | 7.37 | | 1.326 | | 6.67 | | 1.422 | | 5.77 | 1.755 | | 6.20 | | 1.215 |
| 18 | 今年88岁的张大爷，受疝气折磨病情愈加严重，在常州一医院的医生建议下找到该院胃肠外科的卢医生就诊。“我们四处求医，医生都不敢医治，说年龄太高了，就一直拖着。眼看着父亲因为病痛夜不能寐，身体也越来越差。实在没办法了，才辗转到了这家医院。”张大爷的家属说道。 该医院没有拒绝他们。卢主任了解到老人曾装过心脏支架且一直有慢性咳嗽，耐心讲解了手术的危险性。回家后，张大爷的家人看着病情愈发严重的老人，经过反复商量，还是决定冒险一搏。 考虑到老人的特殊情况，卢主任为老人的手术亲自操刀。卢医生擅长胃肠道肿瘤的腹腔镜手术、腹壁切口疝和腹股沟疝的腹腔镜手术，他告诉记者：“虽然患者已经是88岁的高龄，而且身体还有其他病症，但是作为一名医生，我们不能将这样的患者拒之门外。我们医院最近还收治了一位身患胃癌的92岁老人，在进行各项检查和评估之后，我们为老人进行了手术，整个手术持续了三四个小时，十分成功，老人  在手术结束后的第二天就可以下床了。” “幸好遇到了卢主任，他对我父亲的病情进行了仔细地诊疗，又亲自操刀手术，现在我父亲恢复良好，真的是太感谢卢主任的高超医术及全体医护人员的悉心照料！”张大爷的家属感谢之情溢于言表。  Uncle Zhang, who is 88 years old this year, is suffering from hernias more and more seriously. With the advice of a doctor from a hospital in Changzhou, he found Dr. Lu, the gastrointestinal surgeon of the hospital. "We went to see a doctor everywhere, but the doctors didn't dare to treat us. They said that they were too old, so they kept dragging on. As my father couldn't sleep at night because of his illness, his health getting worse and worse. So I really had no choice but to go to this hospital." Uncle Zhang's family said.  The hospital did not reject them. Director Lu learned that the old man had been fitted with heart stents and had been suffering from chronic cough, so he patiently explained the danger of the operation. After returning home, Uncle Zhang's family looked at the old man who was getting worse and worse, and after much discussion, they decided to take a risk.  Taking into account the special circumstances of the elderly, Director Lu personally operated the operation for the elderly. Dr. Lu is good at laparoscopic surgery for gastrointestinal tumors, abdominal incisional hernia and inguinal hernia, He told reporters: "Although the patient is 88 years old and has other diseases, as a doctor, we can't turn away such patients. Our hospital recently admitted a 92 year old man suffering from gastric cancer. After various examinations and assessments, we operated on the old man. The operation lasted for three or four hours and was very successful. The old man can get out of bed the next day after the operation."  "Fortunately, I met Director Lu, who carefully diagnosed and treated my father's illness and performed surgery himself. Now my father is recovering well. I really appreciate Director Lu's superb medical skills and the care of all medical staff!" Uncle Zhang's family expressed their gratitude. | | 311 | | | | | 7.53 | | 1.106 | | 6.87 | | 1.634 | | 6.20 | 1.540 | | 6.20 | | 1.215 |
| 19 | 11月25日，60岁的蔡女士带着保存了33年的随访信，辗转找到了罗医生。“对医院和医生，我只想说谢谢你们！”一见面，蔡女士紧紧握住了罗医生的手，回忆起当年的情形。 1981年，蔡女士来到湘雅医院，被诊断为贲门失弛缓症，随后在胸外科接受了手术。在医护人员的精心照料下，手术后两周左右，她顺利康复出院。 1987年，蔡女士收到了一封来自湘雅医院胸外科的信件，信中详细地询问了她术后有无吞咽困难，有无胸窝部疼痛，有没有继续在当地医院检查，术前术后体重是否有差别；并建议她复诊。 “收到这封信的时候，我感到既惊讶又感动，觉得湘雅的医生非常负责，6年过去了还牵挂着患者的情况。”蔡女士回忆，当时身体恢复地很好，这封信被她一直保留了下来。 今年，出现了当年类似的症状，蔡女士再次来到湘雅医院，经确诊并非旧病复发。一桩心事放下了，但她还想找到当年那位寄出随访信的  医生，当面表达感谢。经过多方寻找，确认寄出这封随访信的是罗医生。 看到自己初入医院时写下的熟悉字迹，罗医生感慨万分：“曾经有一位医学大师说过，一个杰出的医生，随访记录应该从手术前开始一直做到坟墓。虽然术后随访工作做起来繁复，但能切实提高我们的医疗质量。我们的一切付出都是值得的。”  On November 25, Ms. Cai, 60, found Dr. Luo with a follow-up letter that she had kept for 33 years. "To hospitals and doctors, I just want to say thank you!" When we met, Ms. Cai firmly held Dr. Luo's hand and recalled the situation in those years.  In 1981, Ms. Cai came to Xiangya Hospital, where she was diagnosed with achalasia, and then underwent surgery in the thoracic surgery department. With the careful care of the medical staff, she recovered and left the hospital about two weeks after the operation.  In 1987, Ms. Cai received a letter from the Department of Thoracic Surgery of Xiangya Hospital, in which she asked in detail whether she had difficulty swallowing, chest and fossa pain after surgery, whether she continued to check in the local hospital, and whether there was any difference in weight before and after surgery; She was advised to return to the hospital.  "When I received this letter, I was both surprised and moved. I felt that Xiangya's doctor was very responsible and worried about the patient's situation after six years." Ms. Cai recalled that she was recovering well at that time, and she kept this letter.  This year, similar symptoms occurred in the same year. Ms. Cai came to Xiangya Hospital again, and was confirmed not to have relapsed. A matter of concern has been put down, but she still wants to find the doctor who sent the follow-up letter and express her gratitude to him face to face. After many searches, it was confirmed that the follow-up letter was sent by Dr. Luo.  Seeing the familiar handwriting he wrote when he first entered the hospital, Dr. Luo sighed with emotion: "Once a medical master said that an outstanding doctor should keep a follow-up record from the surgery to the tomb. Although the follow-up work after surgery is complicated, it can actually improve our medical quality. All our efforts are worthwhile." | | 322 | | | | | 7.68 | | 1.351 | | 7.19 | | 1.276 | | 5.97 | 1.888 | | 6.00 | | 1.483 |
| 20 | 3月17日凌晨3时30分，中国第22批赴刚果（金）维和医疗分队的接诊台响起一阵急促的电话铃声，电话那头的声音:“现机场有一名危重患者，板状腹，持续剧烈腹胀腹痛12小时，预计45分钟后送到你院，请做好一切急救准备。” 患者危在旦夕的消息，牵动了全队人员的心。医护技人员迅速赶到各自岗位，快速准备。当病房外荷枪实弹的两辆护卫车护送着虚弱的患者入院时，病区内灯火通明，各检查室内的医护人员早已就绪。 抢救刻不容缓。医生在最短时间内明确诊断，抢救小组立即行动。手术结束已经是凌晨7时30分，但医护人员顾不上休息，就共商后续治疗方案，针对可能出现的病情变化，制定应急预案。他们每日查体，了解阳性体征、在床前详细询问患者的症状，针对患者出现的疼痛及可能出现的吻合口瘘的问题逐一提出对策。 护理组9名护士来自国内不同专科，为实施精准护理，针对术后呼吸道管理及并发症的预防进行详细讨论。术后患者腰部疼痛，护士们帮助他进行腰骶部按摩，并告知长期使用止痛药物容易成瘾的副作用，患者感激地竖起大拇指，配合护士进行疼痛评估。术后第三天，患者担心胃管拔除后会声音嘶哑，护士对其进行耐心解释，消除顾虑。经过及时的功能锻炼和进食指导使得患者恢复了体力，第7天，他带着感激之情依依不舍地向医生护士们道别出院。 　　在给医护人员意见栏中，他这样写道：“感谢全体医生护士们，是你们精湛的医疗技术和优质的护理服务给了我第二次生命。”  At 3:30 a.m. on March 17, the reception desk of the 22nd batch of Chinese peacekeeping medical teams to the Democratic Republic of the Congo (DRC) rang a rapid phone call. The voice at the other end of the phone said, "There is a critically ill patient at the airport, with a flat abdomen, who has been suffering from severe abdominal distension and abdominal pain for 12 hours. It is estimated that he will be sent to your hospital in 45 minutes. Please make all emergency preparations."  The news that the patient was in imminent danger affected the whole team. The medical and nursing technicians rushed to their posts and made rapid preparations. When the weak patients were escorted to the hospital by two escort vehicles with guns outside the ward, the lights in the ward were bright and the medical staff in each examination room were ready.  Rescue is urgent. The doctor shall make a clear diagnosis in the shortest time, and the rescue team shall take immediate action. The operation ended at 7:30 a.m., but the medical staff did not have time to rest, so they discussed the follow-up treatment plan and formulated an emergency plan for possible changes in the condition. They checked the body every day to understand the positive signs, asked the patient's symptoms in detail before the bed, and proposed countermeasures one by one for the patients' pain and possible anastomotic leakage.  The nine nurses in the nursing group came from different specialties in China. In order to implement precise nursing, the management of respiratory tract and the prevention of complications after surgery were discussed in detail. After the operation, the patient suffered from lumbar pain. The nurses helped him to massage the lumbosacral region and told him about the side effects of long-term use of painkillers, which led to addiction. The patient thumbed up gratefully and cooperated with the nurse to assess the pain. On the third day after the operation, the patient was worried that his voice would be hoarse after the gastric tube was removed. The nurse explained patiently to him to eliminate his concerns. After timely functional exercise and feeding guidance, the patient recovered his strength. On the seventh day, he reluctantly said goodbye to doctors and nurses with gratitude.  In the comment column for the medical staff, he wrote: "Thank all the doctors and nurses. It is your superb medical technology and high-quality nursing service that gave me my second life." | | 412 | | | | | 7.70 | | 1.119 | | 6.60 | | 1.812 | | 6.07 | 2.016 | | 6.30 | | 1.579 |
| 21 | 11月24日上午，秀山县人民医院消化内科病区上演了温馨的一幕：即将痊愈出院的患者杨女士及其女儿手捧一面写着“ 医术高明，手到病除”字样的锦旗，再三感谢该科宋主任、任护士长及科室全体医护人员对她的精心治疗和悉心护理，让她重拾了新生的希望。 原来，57岁的患者杨女士2019年11月无明显诱因出现上腹部疼痛，不明原因频繁呕吐导致乏力、纳差，曾先后多次到各医院住院治疗，在外近一年的求医，患者病情日渐加重，家庭经济也不堪重负。2020年11月5日其家属抱着完全没有希望，只是输点“营养液”的心态来到该医院消化内科就医。患者病情危重，机体状况极差。经过两次全科会诊，考虑到患者年龄较大，最后确定治疗方案：每天记录患者的进食、意识、睡眠等情况，主管的简医生和吴医生每天坚持饮食、心理及药物指导，慢慢的病人能进食了，呕吐的次数减少了，腹痛减轻了，夜间睡眠时间增加了，病人能站起来了......经过20天的治疗，奇迹般地重生，患者及家属都幸福的笑起来了。 患者的女儿动情地说：感谢医生高超的医术，感谢医护人员全程细致、关爱、精湛的治疗，让妈妈重拾了生命，让她们全家又有了一个完整、温馨的家。 “这一面锦旗是患者对消化内科全体医护人员的赞许，是对每一位医务人员一点一滴付出的认可和肯定，更是对我们今后工作的一种鞭策，让我们牢记医者最初的誓言：健康所系，性命相托，今后我们将更加尽心做好工作，全心全意为患者服务。”宋主任表示。  On the morning of November 24, the Department of Gastroenterology of Xiushan County People's Hospital staged a warm scene: Ms. Yang and her daughter, who were about to recover and leave the hospital, held a banner with the words "skillful medical skills, get rid of the disease with ease" in their hands, and repeatedly thanked the director of the department, the head nurse and all the medical staff of the department for her meticulous treatment and care, so that she regained the hope of a new life.  It turned out that Ms. Yang, a 57 year old patient, had no obvious inducement of epigastric pain in November 2019. She vomited frequently for unknown reasons, which led to fatigue and poor appetite. She had been hospitalized in various hospitals for many times. She had been seeking medical treatment abroad for nearly a year. The patient's condition was getting worse, and her family economy was also overwhelmed. On November 5, 2020, his family members came to the digestive department of the hospital for medical treatment with the mentality that they had no hope at all, but only had to inject some "nutrient solution". The patient's condition is critical and the body condition is very poor. After two general consultations, considering the patient's age, the treatment plan was finally determined: record the patient's eating, consciousness, sleep and other conditions every day, and Dr. Jian and Dr. Wu in charge insisted on diet, psychological and drug guidance every day. Slowly, the patient could eat, the number of vomiting was reduced, abdominal pain was alleviated, the night sleep time was increased, and the patient could stand up...... After 20 days of treatment, the patient was miraculously reborn, The patients and their families all laughed happily.  The patient's daughter said affectionately: Thank the doctors for their superb medical skills, and thank the medical staff for their meticulous, caring and exquisite treatment throughout the whole process, so that the mother can regain her life and their family can have a complete and warm home.  "This banner is the praise of the patients to all medical staff in the Department of Gastroenterology, the recognition and affirmation of every medical staff's efforts bit by bit, and also a spur to our future work. Let us remember the original oath of the doctors: health is everything, life is everything, and we will work harder and serve the patients wholeheartedly in the future." Director Song said. | | 405 | | | | | 7.97 | | 1.159 | | 7.10 | | 1.668 | | 5.63 | 2.251 | | 6.30 | | 1.557 |
| 22 | 从寻求治疗到被送进方舱医院，历经生死之后，一位康复出院的武汉商人，重新返回了医院，寻找在接受治疗期间帮助过他的杨护士。2月28日，央视新闻联播播出了一则《寻找杨护士》的特写，该患者在接受采访中称，他和父亲及多位家人在今年1月下旬感染了新冠病毒，由于初期所有人对新型冠状病毒还不了解，对他来说，比病毒更可怕的，是心里的恐惧。 在社区帮助下，他和父亲被接到了该医院治疗。刚进入医院，他就遇到了江西的一名护士，“当时他问我小伙子你有多大，我说我24，她说小伙子你不要怕，你很年轻，相信自己一定没事的，我们会竭尽全力救助患者。” 简单的几句话，瞬间化解了该患者心里的恐慌，他说，医生们都在冒着生命危险在救治患者。在康复出院后，他和很多帮助过自己的医护人员合影，遗憾的是，没能再见到杨护士。 2月23日，在江西医疗队的帮助下，该患者终于找到了这名来自江西省南昌市新建区人民医院的杨护士。出院患者重返医院感谢护士的一幕，感动并激励了无数抗疫防疫一线的患者和医护人员。3月3日，江西省南昌市委向全市发出了关于号召向杨同志学习的决定。  From seeking treatment to being sent to the shelter hospital, after life and death, a Wuhan businessman who was discharged from hospital after rehabilitation returned to the hospital, looking for treatment.  Nurse Yang helped him during the period. On February 28, CCTV News Network broadcast a feature of "Looking for Nurse Yang". In an interview, the patient said that he, his father and many family members were infected with COVID-19 in late January this year. Since everyone did not know about novel coronavirus at the beginning, what was more terrible for him than the virus was his fear.  With the help of the community, he and his father were treated in the hospital. As soon as he entered the hospital, he met a nurse in Jiangxi. "When he asked me how old you were, I said I was 24, and she said you should not be afraid, young man. You are very young, believe in yourself. We will do our best to help the patients." In a few simple words, the panic in the patient's heart was instantly resolved. He said that doctors were risking their lives to cure the patient. After he recovered from the hospital, he took a group photo with many medical staff who had helped him. Unfortunately, he did not see Nurse Yang again. On February 23, with the help of Jiangxi medical team, the patient finally found nurse Yang from the People's Hospital of Xinjian District, Nanchang City, Jiangxi Province. The scene of the discharged patients returning to the hospital to thank the nurses touched and inspired countless patients and medical staff on the front line of epidemic prevention. On March 3, Nanchang Municipal Party Committee of Jiangxi Province issued a decision on calling on Comrade Yang to learn from him. | | 296 | | | | | 8.00 | | 1.083 | | 7.10 | | 1.647 | | 6.43 | 1.960 | | 6.40 | | 1.303 |
| 23 | 4月中旬，77岁的张爷爷，因糖尿病史20多年并发多种并发症，进入渝北区人民医院肾内·内分泌科治疗，刚入院时，足部伤口创面大至2X5cm，肉眼可见溃烂分泌物，经医生评估创口恢复至少需要半年以上时间。 高龄的张爷爷，若是血糖控制不稳定，还会使创口的恢复时间更加延长。陈主任和刘护士长针对张爷爷的病情立即召开科室病例讨论会，在治疗的同时加强精心护理，以促使足部伤口早日康复。唐护士勇挑重担，承担起了这份护理责任。 一开始，唐护士每天都要蹲在张爷爷的病床前，反复清洗、消毒、上药，指导家属做日常生活护理，饮食健康教育指导，常常一蹲就是一小时。经过一个多月精心的护理和家属们的积极配合，张爷爷足部的伤口创面有了很好的改善，张爷爷的家属欣喜无比，真诚地送上一封感谢信：“小唐认真、细致、耐心的换药打动了我们，特为小唐点赞！” 张爷爷的康复速度是令人欣喜的，但大家并没有因此而松懈，唐护士更是用心观察、精心护理，就是休息日也不放弃对张爷爷的定时换药及观察记录，指导日常生活及饮食健康护理。 7月初，张爷爷足部的伤口已经从最开始的2x5cm愈合至0.2x0.2cm，于是张爷爷家属又再次给医院写了第二封感谢信。“为小唐点赞，为所有医护人员点赞，因为小唐的精心护理而大大缩短了伤口愈合时间......”  In the middle of April, Grandpa Zhang, 77, was admitted to the Kidney Endocrinology Department of Yubei District People's Hospital for treatment because of his history of diabetes for more than 20 years and multiple complications. When he first entered the hospital, the foot wound was as large as 2X5cm, and festering secretion was visible to the naked eye. According to the doctor's assessment, the wound recovery took at least half a year.  The elderly Grandpa Zhang, if the blood sugar control is not stable, will also make the wound recovery time even longer. Director Chen and head nurse Liu immediately held a department case discussion on Grandpa Zhang's condition, and strengthened careful nursing while treating, so as to promote the early recovery of foot wounds. Nurse Tang bravely shouldered the responsibility of nursing.  At the beginning, Nurse Tang would squat in front of Grandpa Zhang's bed every day, repeatedly cleaning, disinfecting, and applying medicine to guide the family members to do daily life care, diet health education, and often squatting for an hour. After more than a month of careful care and the active cooperation of his family members, Grandpa Zhang's foot wound has been greatly improved. Grandpa Zhang's family members are very happy and sincerely sent a letter of thanks: "We are impressed by Xiao Tang's careful, meticulous and patient dressing change, and I would like to praise him!"  The speed of Grandpa Zhang's recovery was gratifying, but everyone did not relax. Nurse Tang observed carefully and nursed him carefully. Even on the rest day, she did not give up the regular dressing change and observation record of Grandpa Zhang to guide daily life and diet health care.  At the beginning of July, Grandpa Zhang's foot wound had healed from the initial 2x5cm to 0.2x0.2cm, so his family wrote a second letter of thanks to the hospital again. "Praise Xiao Tang and all the medical staff. Thanks to Xiao Tang's meticulous care, the wound healing time has been greatly shortened..." | | 332 | | | | | 8.06 | | 1.029 | | 7.30 | | 1.591 | | 5.91 | 2.037 | | 5.85 | | 1.889 |
| 24 | 自治区新冠肺炎医疗救治定点医院罗医生：“有一封感谢信让我们所有的医护人员印象特别深，也特别感动。她写了4遍，每写1遍她都觉得不是那么的满意，每次都要修改。我就给这个患者说，你的心意我们也感受到了，这也是我们所有医护人员应该做的事情，但这个病人特别执着，对我们也是特别深的触动。” 一名患者在感谢信中写到：“有一次，转病房送我们过去的护士姐姐说她头晕，我让她赶紧坐下来休息一会，她说没关系，我先把你们送上去，当时觉得很心疼……” 罗医生说，写这封信的患者临床诊断为轻型，虽然没有发烧、乏力、咳嗽等症状，但刚入院时，紧张、焦虑、恐惧的情绪还是十分强烈。为了稳定这名患者的情绪，病区专门为她请来心理医生进行心理疏导。医护人员们只要有时间，也会去她的病房陪她聊天，舒解她的心情。罗医生：“我们每次给病人传递的就是我们是你的亲人，有任何需要帮助的，我们都可以做，有任何困难一定要找医护人员，我们一定会尽全力解决困难。” 情绪稳定后，这名患者积极配合治疗，只用了一个疗程就康复出院。她也专门为医护人员送上了祝福。她说：“医护人员，一个医治生命的团队，值得我们歌颂，真心希望你们能够早日回家，与家人团聚。”罗医生：“患者对我们的认可和肯定(也鼓励着我们)，按照国家卫健委第八版新冠肺炎的诊疗方案，给患者进行标准化同质化的治疗，让更多患者痊愈出院。”  Dr. Luo, the designated hospital for medical treatment of COVID-19 in the autonomous region: "There was a thank-you letter that impressed and moved all our medical staff. She wrote it four times, and every time she wrote it, she was not so satisfied. Every time she wrote it, she had to revise it. I told the patient that we also felt your heart, which is what all our medical staff should do, but the patient was very persistent and touched us deeply."  A patient wrote in a thank-you letter: "Once, the sister nurse who transferred us to the ward said that she was dizzy, and I asked her to sit down and rest for a while. She said it was OK. I sent you up first, and I felt very distressed at that time..."  Dr. Luo said that the patient who wrote this letter was clinically diagnosed as mild. Although he had no symptoms such as fever, fatigue and cough, he was very nervous, anxious and afraid when he first entered the hospital. In order to stabilize the patient's mood, the ward specially invited a psychologist for psychological counseling. As long as the medical staff have time, they will go to her ward to chat with her and relieve her mood. Dr. Luo: "Every time we communicate to patients that we are your relatives, we can do whatever we need. If there is any difficulty, we must find medical staff, and we will do our best to solve it."  After emotional stability, the patient actively cooperated with the treatment, and only took one course of treatment to recover and leave the hospital. She also gave her best wishes to the medical staff. She said: "The medical staff, a team that cures life, is worthy of our praise. I sincerely hope that you can go home and get together with your family as soon as possible." Dr. Luo: "The patients' recognition and affirmation of us (also encourages us), according to the eighth edition of the diagnosis and treatment plan of the National Health Commission for COVID-19, we will provide standardized and homogeneous treatment for patients, so that more patients will recover and leave the hospital." | | 364 | | | | | 8.13 | | .885 | | 7.32 | | 1.137 | | 5.87 | 2.172 | | 5.94 | | 1.526 |
| 25 | 安先生的母亲今年75岁，10月17日上午，安先生的母亲准备下楼晾晒被子，不料却在一楼剩下4个台阶的楼梯上踩空摔落，整个身子疼的不能动弹，被送往西安市红会医院就医。 10月25日中午，在心内科、麻醉科等多学科医护人员协作下，欧医生为老人做了肱骨近端骨折切腹内固定术，手术历时1个半小时，十分顺利，医护人员护送老人回到了病房。 然而，刚回到病房不到一小时，危急情况却发生了。老人突然出现血压不稳，创口引流袋有较多出血。欧医生立刻给老人用创口加压止血，使用止血药、紧急补液、输血。并通知马副教授前来会诊协助处理。 傍晚6点多，老人情况稳定了下来，手外科一病区张护士还一直坐在病房内守着老人。记者在采访中得知，她两天两夜没有回家，欧医生因放心不下老人的病情，推掉了外出开会。 安先生母亲苏醒后说的第一句话是“感谢医生护士，感谢你们所有的人对我的帮助，你们的团队救了我。” 欧医生说，医患配合才能取得最好的治疗效果。在采访中得知，经常有陌生人给欧医生来电话询问一些疾病治疗的问题和建议，欧医生认为患者来电是对他医术的信任，利用自己的专业知识答疑解惑只是举手之劳，当然不能辜负患者的信任。心同此心，理同此理。每个人都要有健康的心态，正确对待生老病死，作为患者家属要理解医护人员，相信他们可以“治好病”，积极配合治疗。  Mr. An's mother is 75 years old this year. On the morning of October 17, Mr. An's mother was going to go downstairs to dry the quilts, but she fell down on the stairs with four remaining steps on the first floor. Her whole body was too painful to move, and she was sent to Xi'an Red Cross Society Hospital for medical treatment.  At noon on October 25, with the cooperation of multidisciplinary medical staff such as cardiology department and anesthesiology department, Dr. Ou performed abdominal internal fixation of proximal humerus fracture for the elderly. The operation lasted 1.5 hours and was very successful. The medical staff escorted the elderly back to the ward.  However, less than an hour after returning to the ward, a critical situation occurred. The old man suddenly had unstable blood pressure, and there was more bleeding in the wound drainage bag. Doctor Ou immediately put pressure on the wound to stop bleeding, and used hemostatic drugs, emergency rehydration and blood transfusion. Associate Professor Ma was also notified to come for consultation and assistance.  At more than 6 o'clock in the evening, the old man's condition stabilized. Nurse Zhang of No. 1 Hand Surgery Ward has been sitting in the ward guarding the old man. The reporter learned in the interview that she did not go home for two days and two nights. Doctor Ou was worried about the old man's condition and pushed her away from the meeting.  The first sentence Mr. An's mother said after she woke up was "thank doctors and nurses, thank all of you for helping me, and your team saved me."  Doctor Ou said that only with the cooperation of doctors and patients can the best therapeutic effect be achieved. In the interview, I learned that strangers often call Dr. Ou to ask him some questions and suggestions about disease treatment. Dr. Ou believes that the patient's call is a trust in his medical skills. Using his professional knowledge to answer questions and solve problems is just a matter of hands. Of course, we should not disappoint the patient's trust. The heart is the same as the heart, and the reason is the same. Everyone should have a healthy attitude and treat life, old age, illness and death correctly. As the family members of patients, they should understand the medical staff, believe that they can "cure the disease" and actively cooperate with the treatment. | | 404 | | | | | 8.19 | | 1.376 | | 7.45 | | 1.690 | | 6.26 | 2.065 | | 6.94 | | 1.340 |
| 26 | A special gift was given to the medical staff of the Fifth Affiliated Hospital. What surprised them was that they "turned" into the person in the picture. They stood vividly revealed on the paper, which was both interesting and lovely, and they were warmed when they saw it... This gift came from a small patient. In addition to drawing a cartoon by hand, she also attached a letter of thanks written in her own handwriting. What's the story here?  It is understood that Xiao Xi, a small patient, is 12 years old this year. On September 28 this year, Xiao Xi was sent to the Fifth Hospital of Zunyi Hospital for sudden abdominal pain, and was diagnosed as acute suppurative appendicitis after examination. In the afternoon, the pediatric surgery department of the hospital performed appendectomy for her, and she recovered well. On October 3, Xiao Xi recovered and was discharged from hospital.  Xiao Xi gave this carefully prepared gift to doctors and nurses when they came back for a further visit. Her mother said that the gift expressed Xiao Xi and her family's sincere thanks to the medical staff. It can be seen from the cartoon that during the hospitalization, Xiao Xi carefully captured every moment of getting along with doctors and nurses. From first acquaintance to acquaintance, she used a brush to record every unforgettable scene in the ward. At the end of the cartoon, Xiao Xi also attached a letter of thanks. Every sentence of childish words is full of sincerity. At the end of the letter, she did not forget to remind her younger brothers and sisters to solve their personal problems（marriage） earlier. After receiving such a special gift, the medical staff of the hospital felt warm and moved. Nurse Huang was the most mentioned by Yingxi in the letter.  Nurse Huang said: "Colleagues in the department were moved when they received the gift from Xiao Xi. Thank her for gently recording the details and stories of our relationship. This gift is also very meaningful to me. This year is my first year in medical care. Being so gently recognized has strengthened my determination to stick to my post. I hope there will be more warm stories between doctors and patients, and we will treat each patient more attentively." | | 385 | | | | | 8.20 | | .997 | | 7.57 | | 1.547 | | 6.60 | 1.993 | | 6.83 | | 1.533 |
| 27 | On the afternoon of 19th, 31 severe patients with COVID-19 recovered from the West Hospital of Wuhan Union Medical College Hospital. "Thank you, doctor!" "Thank you!" Outside the inpatient building, many cured people praised the medical staff.  Mr. Chen is one of these healers. Unlike others, he holds a letter of thanks in his hand. "On the morning of the 8th, I got up and heard that I was going to leave the hospital. I wrote this letter of thanks excitedly for more than an hour. Before coming to the hospital, I was a critically ill patient, and now I am reborn. I thank the medical staff from the bottom of my heart."  Mr. Chen, 47 years old, felt a little feverish on January 25, but his symptoms were not serious at first. After having a fever on January 27, he went to the hospital late at night for examination because he was worried about infection. Later, they began to isolate the community and hotels, and during the isolation, they had a fever of 39.5 degrees. On February 5, Mr. Chen was admitted to the hospital. "I had a fever for six or seven days, but I couldn't endure it. I was very disappointed and helpless at that time. Then I came here for help, and I was admitted."  Mr. Chen did not know why he was infected. He recalled that when he was seriously ill, he could not stand, had difficulty breathing, and could see blood when he coughed in the morning. At this time, it was the medical staff who comforted him and cheered him on the hospital bed. "They sent the meal to me and said that you would have resistance if you ate it."  The palm and heart of Mr. Chen's hand were filled with names, and he said these were the medical staff he would like to thank. He also read his letter of thanks: "When the epidemic is fully successful, let go of your tired bodies... At that time, the people of Wuhan will be full of enthusiasm and gratitude to let you feel the prosperity and beauty of Wuhan." | | 356 | | | | | 8.37 | | .928 | | 7.50 | | 1.432 | | 6.80 | 1.990 | | 6.50 | | 1.456 |
| 28 | At more than 3:00 p.m. on February 11, 2020, there were many media waiting outside the square outside the shelter hospital. On the seventh day of receiving patients in Wuchang Fangcang Hospital, 28 convalescents were about to leave here. From the day when the tent hospital was set up in the open space outside the shelter hospital on February 4, the Shanghai National Emergency Medical Rescue Team started the fight against the epidemic in Wuhan shelter hospital. The shelter hospital has solved the problem of admission of mild patients with COVID-19, but it has also brought great challenges to the work of medical staff.  On the first night of the admission of patients to Wuchang Fangcang Hospital, Dr. Yang, the leader of the medical team of Huashan Hospital, met a patient. The patient wore a mask and said to the medical staff, "Stay away from me, I will infect you. Thank you!" The unknown fear before seeing the patient vanished instantly. Dr. Yang said, "At that moment, I thought everyone was ordinary. The difference was that they were unfortunately infected. We should give them more care." Patient Cai said: "The medical team came from all directions to support us. They were very enthusiastic and careful. I can only thank them!" The patients can truly feel the contributions of medical workers. They heal the pain and comfort the soul. It is hoped that in the near future, patients will be able to walk out of this life ark in a healthy way and start a new journey in their lives. | | 260 | | | | | 8.39 | | 1.174 | | 7.58 | | 1.628 | | 6.45 | 2.063 | | 6.94 | | 1.672 |
