# Supplementary material for: Effect of doctor–patient news-induced moral judgments on pain empathy for doctors and patients in China
Source: Front Neurosci. 2022 Nov 23;16:1037659. doi: 10.3389/fnins.2022.1037659 (PMC9726916; doi:10.3389/fnins.2022.1037659)
Supplement: Supplementary file 2 [file Data_Sheet_2.docx]

**Appendix B**

**pain and non-pain pictures**

| Num | Type | Exercise/Formal experiment | Picture |
| --- | --- | --- | --- |
| 1 | non-pain | Exercise experiment | 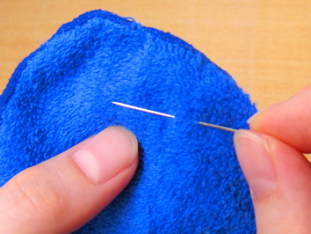 |
| 2 | pain | Exercise experiment | 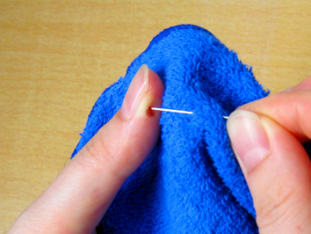 |
| 3 | non-pain | Exercise experiment | 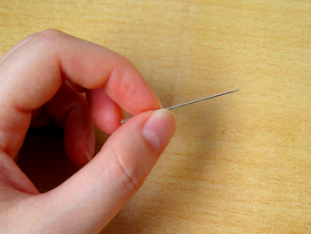 |
| 4 | pain | Exercise experiment | 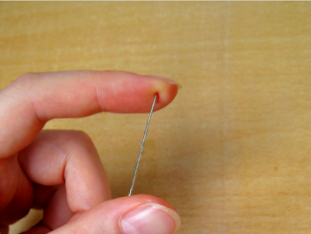 |
| 5 | non-pain | Exercise experiment | 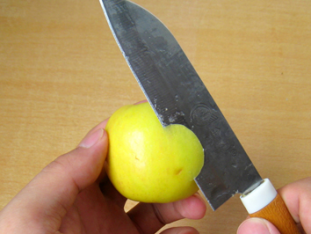 |
| 6 | pain | Exercise experiment | 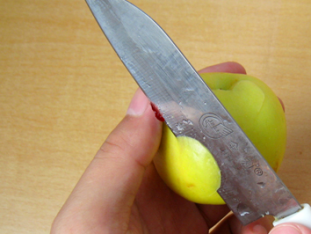 |
| 7 | non-pain | Exercise experiment | 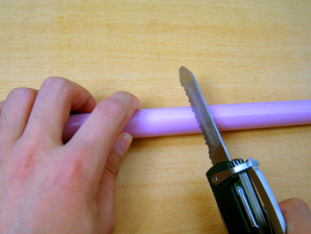 |
| 8 | pain | Exercise experiment | 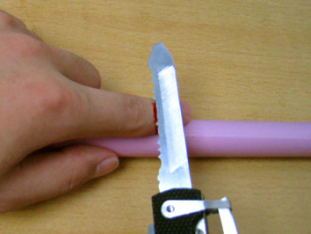 |
| 9 | non-pain | Exercise experiment | 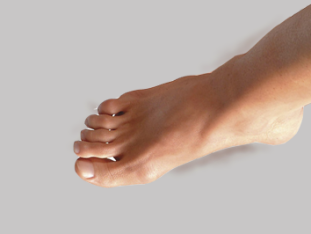 |
| 10 | pain | Exercise experiment | 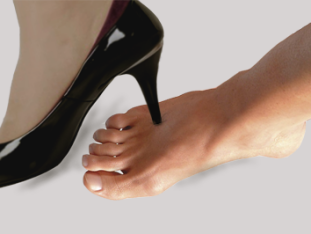 |
| 11 | non-pain | Exercise experiment | 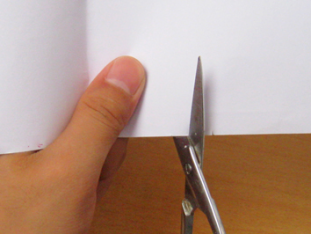 |
| 12 | pain | Exercise experiment | 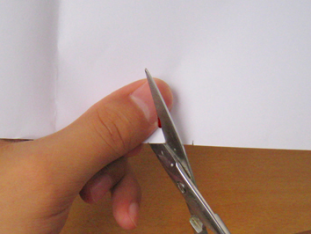 |
| 13 | non-pain | Exercise experiment | 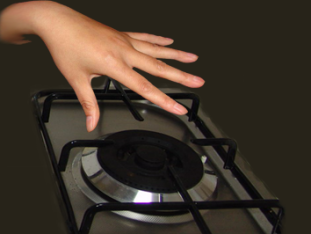 |
| 14 | pain | Exercise experiment | 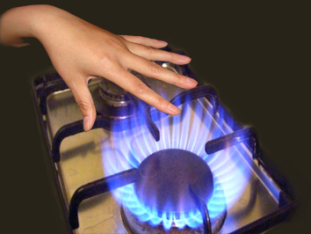 |
| 15 | non-pain | Exercise experiment | 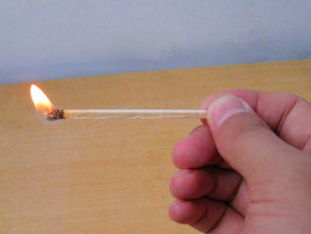 |
| 16 | pain | Exercise experiment | 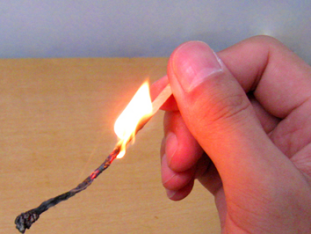 |
| 17 | non-pain | Exercise experiment | 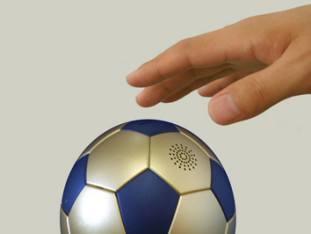 |
| 18 | pain | Exercise experiment | 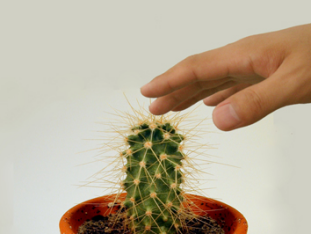 |
| 19 | non-pain | Exercise experiment | 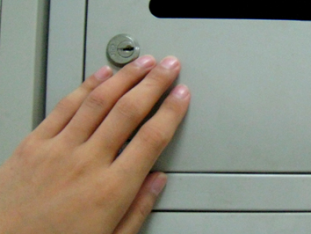 |
| 20 | pain | Exercise experiment | 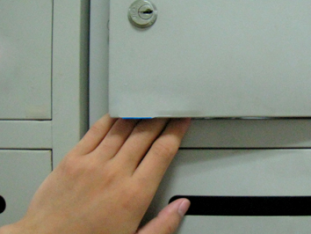 |
| 21 | non-pain | Exercise experiment | 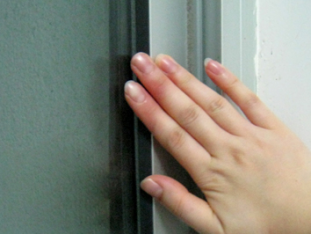 |
| 22 | pain | Exercise experiment | 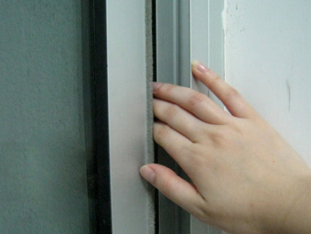 |
| 23 | non-pain | Exercise experiment | 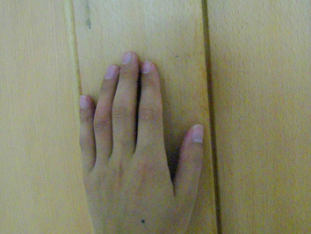 |
| 24 | pain | Exercise experiment | 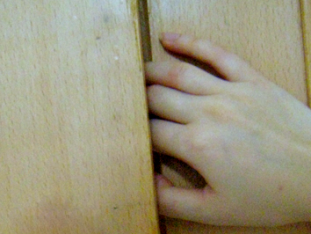 |
| 25 | non-pain | Formal experiment | 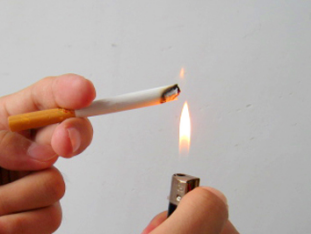 |
| 26 | pain | Formal experiment | 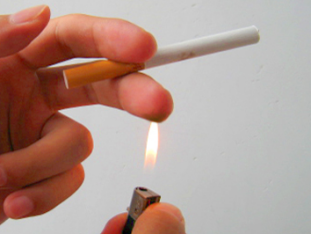 |
| 27 | non-pain | Formal experiment | 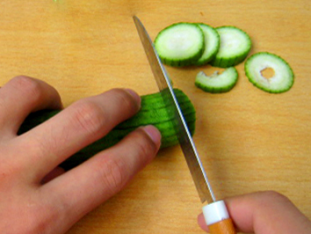 |
| 28 | pain | Formal experiment | 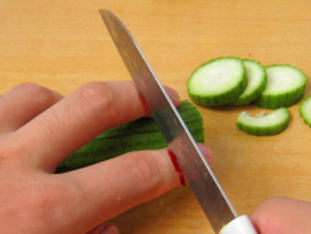 |
| 29 | non-pain | Formal experiment | 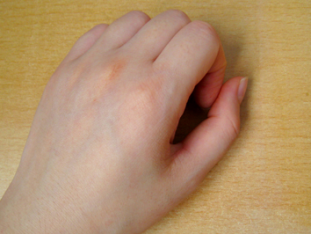 |
| 30 | pain | Formal experiment | 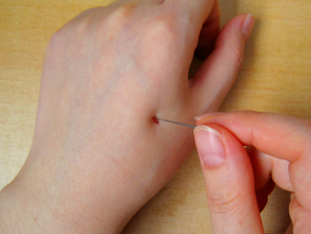 |
| 31 | non-pain | Formal experiment | 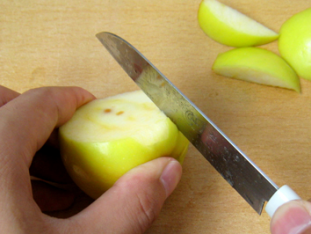 |
| 32 | pain | Formal experiment | 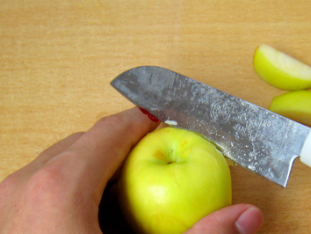 |
| 33 | non-pain | Formal experiment | 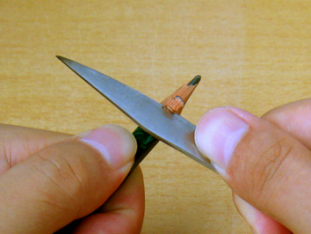 |
| 34 | pain | Formal experiment | 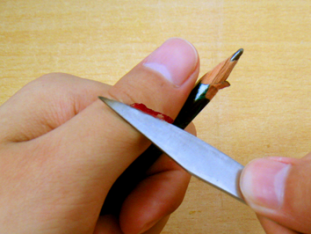 |
| 35 | non-pain | Formal experiment | 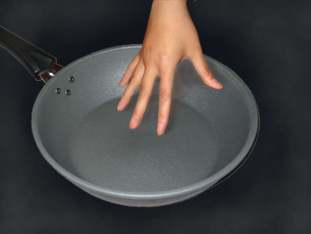 |
| 36 | pain | Formal experiment | 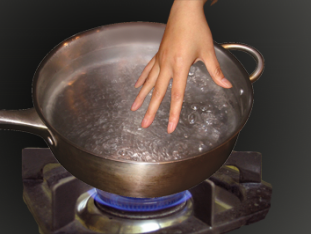 |
| 37 | non-pain | Formal experiment | 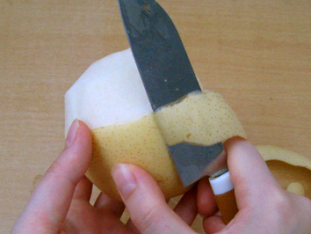 |
| 38 | pain | Formal experiment | 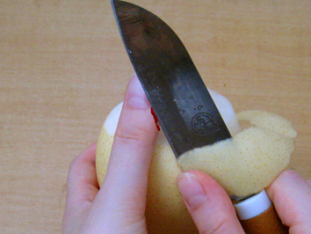 |
| 39 | non-pain | Formal experiment | 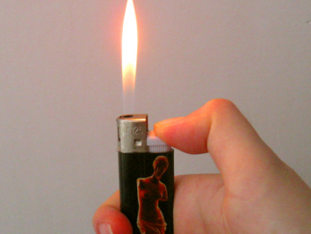 |
| 40 | pain | Formal experiment | 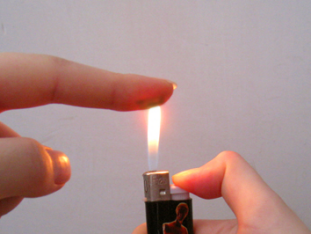 |
| 41 | non-pain | Formal experiment | 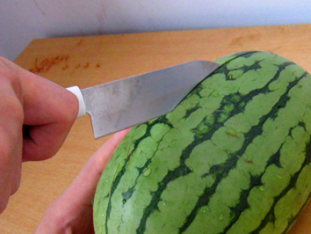 |
| 42 | pain | Formal experiment | 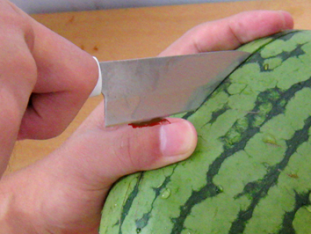 |
| 43 | non-pain | Formal experiment | 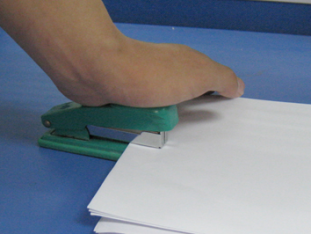 |
| 44 | pain | Formal experiment | 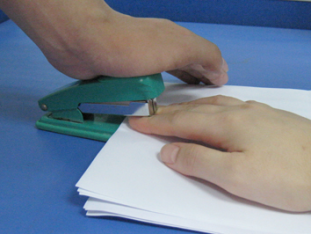 |
| 45 | non-pain | Formal experiment | 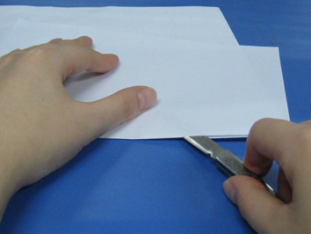 |
| 46 | pain | Formal experiment | 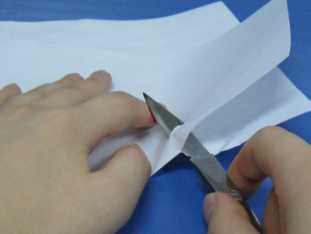 |
| 47 | non-pain | Formal experiment | 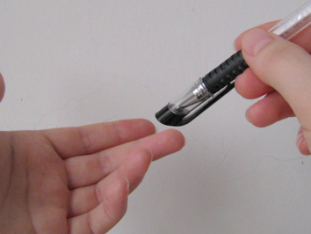 |
| 48 | pain | Formal experiment | 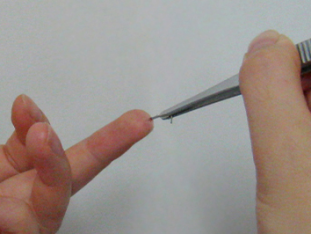 |
| 49 | non-pain | Formal experiment | 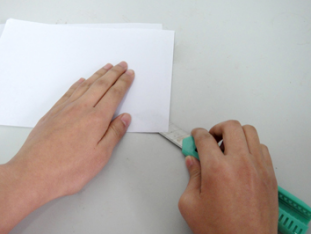 |
| 50 | pain | Formal experiment | 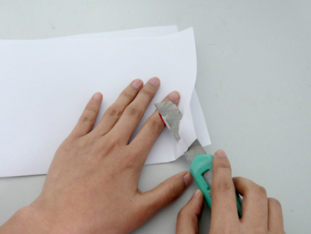 |
| 51 | non-pain | Formal experiment | 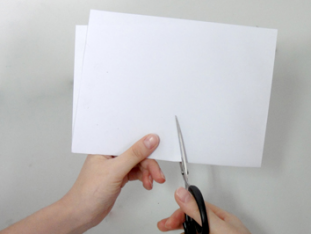 |
| 52 | pain | Formal experiment | 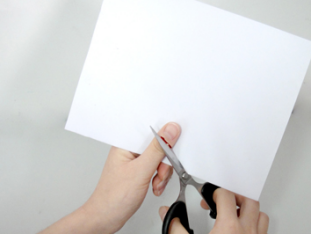 |
| 53 | non-pain | Formal experiment | 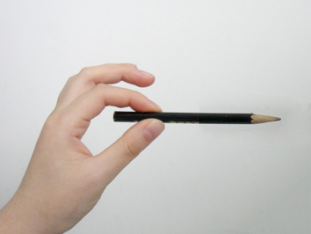 |
| 54 | pain | Formal experiment | 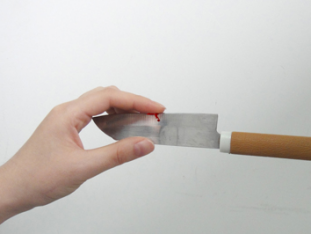 |
| 55 | non-pain | Formal experiment | 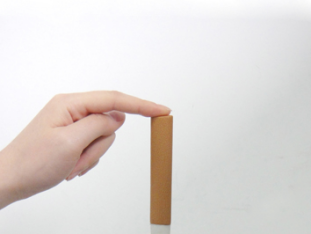 |
| 56 | pain | Formal experiment | 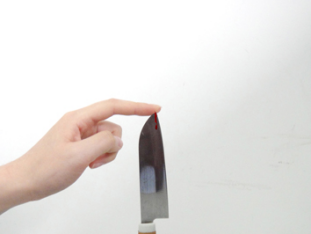 |
| 57 | non-pain | Formal experiment | 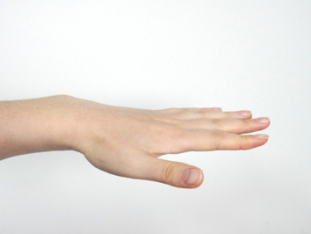 |
| 58 | pain | Formal experiment | 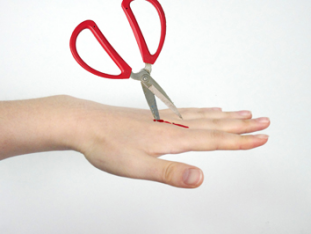 |
| 59 | non-pain | Formal experiment | 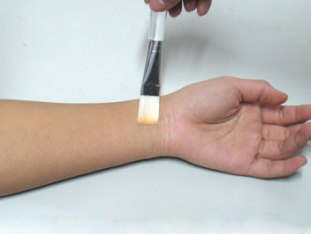 |
| 60 | pain | Formal experiment | 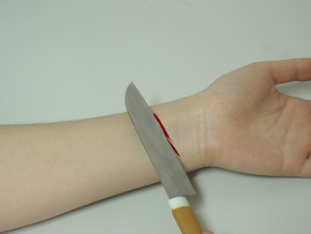 |
| 61 | non-pain | Formal experiment | 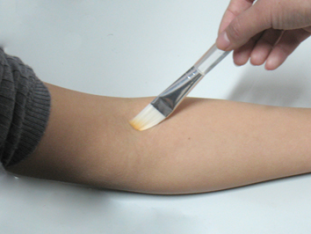 |
| 62 | pain | Formal experiment | 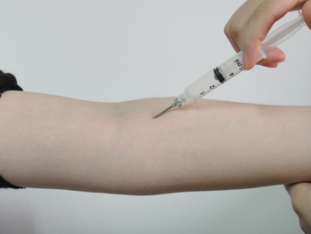 |
| 63 | non-pain | Formal experiment | 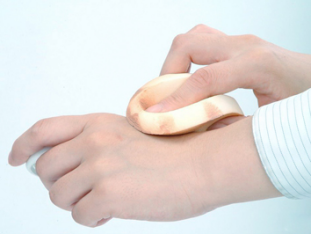 |
| 64 | pain | Formal experiment | 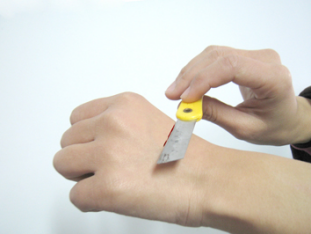 |
| 65 | non-pain | Formal experiment | 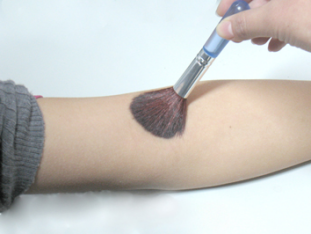 |
| 66 | pain | Formal experiment | 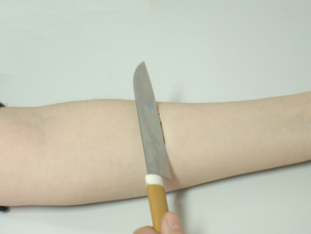 |
| 67 | non-pain | Formal experiment | 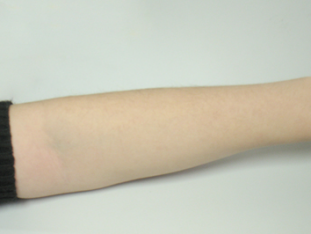 |
| 68 | pain | Formal experiment | 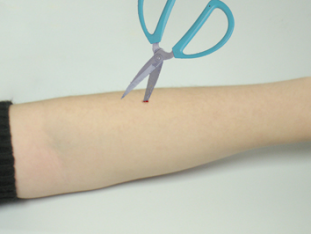 |
| 69 | non-pain | Formal experiment | 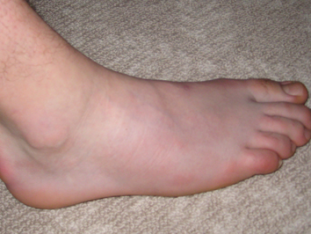 |
| 70 | pain | Formal experiment | 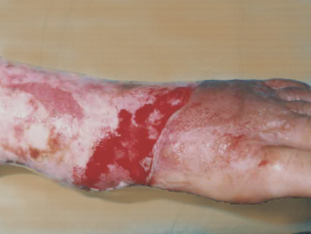 |
